# Strong influence of behavioral dynamics on the ability of testing and treating HIV to stop transmission

**DOI:** 10.1038/srep09467

**Published:** 2015-04-22

**Authors:** Christopher J. Henry, James S. Koopman

**Affiliations:** 1 Department of Epidemiology, University of Michigan, 1415 Washington Heights, Ann Arbor, MI 48109-2029, USA

## Abstract

Choosing between strategies to control HIV transmission with antivirals requires understanding both the dynamics affecting those strategies' effectiveness and what causes those dynamics. Alternating episodes of high and low contact rates (episodic risk) interact with increased transmission probabilities during early infection to strongly influence HIV transmission dynamics. To elucidate the mechanics of this interaction and how these alter the effectiveness of universal test and treat (UT8T) strategies, we formulated a model of UT8T effects. Analysis of this model shows how and why changing the dynamics of episodic risk changes the fraction of early transmissions (FET) and the basic reproduction number (*R*_0_) and consequently causes UT8T to vary from easily eliminating transmission to having little effect. As the length of risk episodes varies from days to lifetimes, FET first increases, then falls. Endemic prevalence varies similarly. *R*_0_, in contrast, increases monotonically and is the major determinant of UT8T effects. At some levels of episodic risk, FET can be high, but eradication is easy because *R*_0_ is low. At others FET is lower, but a high *R*_0_ makes eradication impossible and control ineffective. Thus changes in individual risk over time must be measured and analyzed to plan effective control strategies with antivirals.

The ultimate goal of Treatment as Prevention (TasP), the complete elimination of HIV transmission, will be achieved if the number of transmissions made by a “typical” infected individual over the course of his infection can be reduced to less than one^1^. There are three major factors that affect how difficult it is to achieve this goal through Universal Test and Treat (UT8T): (1) The series of steps that must be traversed from diagnosis to viral suppression (the treatment cascade), (2) the fraction of transmissions that occur early in the transmitter's infected period (which is determined by both biological and social factors), and (3) the basic reproduction number of the virus within the population of interest (which is also affected by both biological and social factors). The basic reproduction number (*R*_0_) and the fraction of early transmissions relate to two dimensions of difficulty in achieving elimination through UT8T: The basic reproduction number is closely related to the fraction of transmissions that must be prevented through diagnosis and treatment in order to achieve elimination (which is 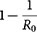), and early transmissions are more difficult to prevent with UT8T than later ones.

The importance of the treatment cascade and the fraction of transmissions from early infection have been extensively discussed, but the importance of the basic reproduction number (*R*_0_), although touched on in some articles^2^,^3^, has received significantly less attention. We show that behavioral dynamics can result in a wide range of *R*_0_s even at a fixed endemic prevalence and a fixed fraction of transmissions from early infection. These variable *R*_0_s, in turn, cause elimination through UT8T to range from easy to realistically impossible. In particular, we explore conditions where, even though a relatively high fraction of transmissions come from early HIV infection (EHI), transmission can still be stopped with a relatively low effective treatment rate, because only a small fraction of transmissions must be interrupted in order to get the system below the endemic threshold of *R*_0_ = 1. This accords with and extends existing modeling work that found that the relative transmissibility during EHI has little effect on the expected reduction in incidence thirty years after the initiation of an antiretroviral therapy (ART) intervention^4^,^5^.

In this paper we seek to make the complex phenomena behind these relationships more understandable, so they can better inform policy decisions. This paper seeks to generate qualitative understandings that will ultimately advance quantitative assessments. Toward this end, we use deterministic compartmental models (DCMs) to model population dynamics. Such models do not account for stochastic features of real-world population dynamics, but they allow for a simpler analysis. Although a DCM does not directly model individuals, it can be understood as the limiting case of an individual-based model of an arbitrarily large population, and flows between compartments can therefore be conceptualized in terms of changes in the states of (large numbers of) individuals. We will make frequent use of this abstraction throughout this paper.

We make our points by examining and explaining the behavior of a model for HIV transmission among men who have sex with men (MSM) closely based on the model developed by Zhang et al.^6^ This model greatly simplifies the treatment cascade and the natural history of infection, while at the same time adding details about temporal patterns of behavior that other models often omit. In order to focus on the effects of these dynamics, we simplify the natural history of infection to a relatively brief early phase (early HIV infection, EHI) followed by a longer chronic phase. In §4.4 of the Supplementary Results, we show that our qualitative results continue to hold if this simplified natural history is replaced with a more realistic one, based on results from a study of HIV transmission in Lilongwe, Malawi^7^.

Similarly, we collapse the treatment cascade into a single parameter measuring the rate at which infected individuals are tested, treated, and rendered permanently non-transmitting, which we refer to as the *effective treatment rate*. Each individual is therefore classified as effectively treated or untreated. The untreated category includes undiagnosed; diagnosed, but untreated; and treated, but not virally suppressed individuals. In addition, all sexual partnerships are treated as instantaneous symmetrical contacts. Although all of these simplifications will affect some of the quantitative results that we obtain, we do not believe that any of them affect our qualitative conclusions. For more details about the model used, see §3.1 of the Supplementary Methods.

The purpose of these simplifications is to highlight the effect that different patterns of contact rates over time can have on the response of transmission systems to universal test-and-treat interventions, even when other aspects of those systems are identical. Our take-home message is that populations can differ with regard to patterns of contact rates in ways that drastically affect the effort required to achieve elimination of ongoing transmission through UT8T. We illustrate this with a simplified model of HIV transmission among MSM that is designed to highlight certain aspects of behavioral complexity. From this model we gain insights that suggest other aspects of real-world transmission systems that could generate similar effects.

## Results

### Characteristics of interest

The primary characteristics of sexual contact patterns that we examine in this paper are *risk heterogeneity* and *episodic risk*. Risk in our model is only determined by contact rates, which are in turn determined by which of two risk groups an individual belongs to. Our key results still hold with more than two risk groups, but for the sake of simplicity, we focus on a model with only two, which we will refer to as the higher risk group and the lower risk group.

In models where individuals always remain in the same risk group, we say there is *static risk heterogeneity*. In models where individuals transition between risk groups, we say there is *episodic risk*.

We model episodic risk by selecting risk groups for individuals newly entering the sexually active population, and then reselecting their risk groups at intervals corresponding to a fixed rate of reselection (*ω*). The probability of being selected to the higher or the lower risk group is always the same. Consequently, the fraction of the population that is in the higher risk group at the disease-free equilibrium is unaffected by whether there is static risk heterogeneity or episodic risk. The average length of an individual's stay in a risk group is therefore determined by both the re-selection rate and the probability of being assigned to that risk group at each point when they are re-randomized to risk groups. Thus, for example, if the probability of being assigned to the high risk group were 0.2, the average stay in the lower risk group would be four times the average stay in the higher risk group if no one left the sexually active population.

Note that this parameterization of the episodic risk formulation is the major way that this episodic risk model differs from the model in Zhang et al.^6^ Instead of rates of transitioning between risk groups, we have this single re-selection rate parameter. This parameterization is chosen because it simplifies the mathematical analysis of the system and makes the origin of episodic risk effects clearer. It is not a causal model of what generates episodic risk, but an abstraction that allows us to make important qualitative observations.

We parameterize differential transmissibility over the course of infection with a parameter (*ζ*) that gives the ratio of per-act transmissibility during early HIV infection (EHI) to per-act transmissibility during later/chronic infection. We will refer to this ratio as the relative transmissibility during EHI.

We focus most of our attention in this paper on these two parameters (*ω* and *ζ*) in order to highlight the interaction of biological and behavioral factors in determining the difficulty of achieving elimination through UT8T, with a particular focus on an aspect of sexual behavior (episodic risk) that has received relatively little attention.

### Homogeneous transmission potentials and episodic risk

To develop theory in a progressive fashion, we begin by considering a behaviorally homogeneous population, and we examine how the basic reproduction number changes when we add first static risk heterogeneity, and then episodic risk. In order to achieve maximum clarity, we first do this with a single-stage SI model, and then show how the results extend naturally to models with multiple stages of disease progression (such as the one used in this paper).

When the population is behaviorally homogeneous, and there is only one stage of infection, the basic reproduction number is simply the product of the (average) contact rate (*χ*), the per-contact transmissibility (*β*), and the average duration of infection (*D*). We will refer to this basic reproduction number, calculated under conditions of behavioral homogeneity, as the *(total) homogeneous transmission potential*, and denote it by *H*:
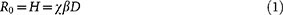
This behavioral homogeneity will be achieved if the “higher” and “lower” risk groups actually have the same contact rate (*χ*) and there is only one transmission probability (*β*) over the entire duration of infection (D).

We elaborate this model by introducing static risk heterogeneity, i.e. by allowing the two risk groups to have different contact rates, but requiring that the re-selection rate be 0, so that individuals stay in the same risk group for their entire sexually active lives. We assume here that risk groups mix proportionately^8^, except where otherwise noted. In §3.6 of the Supplementary Methods, we analyze a model of assortative mixing between risk groups and show that our major qualitative results still hold. Given static risk heterogeneity, the basic reproduction number increases in proportion to the square of the coefficient of variation of contact rates (equation adapted from May and Anderson^9^):
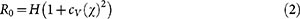
This occurs because members of the higher risk group will have both an elevated propensity to become infected and (once infected) an elevated propensity to transmit. This equation depends on the average contact rate (which determines *H*) and on the coefficient of variation of contact rates, but not on other features of the distribution of contact rates. Consequently, it still holds if the model is extended to have more than two risk groups, or to have a continuous distribution of contact rates.

We now further elaborate the model by introducing episodic risk, i.e. by allowing the re-selection rate to be greater than 0. In this formulation, an individual who is in the higher risk group at the time of infection will now have an elevated propensity to transmit only until their risk group is re-selected. Because the (multiplicative) increase to the basic reproduction number is a result of the same individuals who have an elevated propensity to become infected also having an elevated propensity to transmit, it will therefore only be applied to that fraction of the homogeneous transmission potential which represents transmissions occurring *before* we re-select the contact rate of the transmitter. This is simply the fraction of time infected that is, on average, spent prior to our re-selection of the infected individual's contact rate. We denote that fraction (which we call the *(overall) fraction of heterogeneity effect*) by *ψ*. This leads to the following generalization of equation 2:
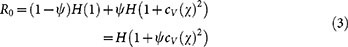


Now we go back to the situation without risk heterogeneity and examine the situation with multiple stages of infection, each with its own per-contact transmission rate. In this case the total homogeneous transmission potential (and therefore the basic reproduction number) will be the sum of the homogeneous transmission potentials from each stage (*H_i_*):
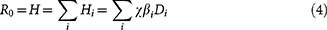
Note that in this case *D_i_* is the average duration of stage *i* for all infected individuals, including (for later stages) those who do not survive long enough to enter stage *i* (i.e., it incorporates the probability of not reaching stage *i* at all). Although the focus of this paper is on a model with only two stages of infection (and therefore the above summation is from i = 1 to i = 2), equation (4) does not depend on that fact, nor do any of the subsequent equations in this section. Consequently, they will still hold if a model with a more realistic natural history of infection is used, as is the case in §4.4 of the Supplementary Results.

Given static risk heterogeneity, the contributions of each stage of infection to the basic reproduction number will be increased by the same multiplicative factor (relative to behavioral homogeneity), and so equation (2) still applies. But when we introduce episodic risk, this will no longer be the case: Because the stages of infection come in order, one after the other, the average fraction of an infected individual's time spent in the *i*-th stage of infection that occurs before his contact rate is re-selected (the *fraction of heterogeneity effect for stage* i, which we will denote *ψ_i_)* will be smaller for larger *i*. In particular, the fraction of heterogeneity effect for chronic infection (*ψ_2_*) will always be less than the fraction of heterogeneity effect for EHI (*ψ_1_*). Therefore, the basic reproduction number will now be:
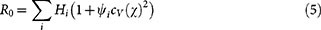
Equation (3) still applies; however, the overall fraction of heterogeneity effect (*ψ*) is no longer simply the fraction of an individual's time infected that occurs prior to the re-selection of his contact rate, because one month of chronic infection and one month of EHI do not contribute equally to the total homogeneous transmission potential (H). Consequently, the overall fraction of heterogeneity effect is now a weighted average of the fractions of heterogeneity effect for each stage:
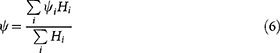


Combining equation (6) with equation (3), we obtain two major implications: First, the more episodic that risk heterogeneity is (i.e. the higher the re-selection rate), the smaller *ψ* will be, and therefore the lower *R*_0_ will be. Second, because the fraction of heterogeneity effect during EHI (*ψ*_1_) will always be greater than the fraction of heterogeneity effect during chronic infection (*ψ*_2_), episodic risk reduces transmissions from EHI less than it reduces transmissions from chronic infection. Consequently, episodic risk raises the fraction of transmissions from early infection while it lowers the basic reproduction number.

The above analysis relates to the potential for transmissions *during exponential growth*. Under these conditions, when the prevalence of infection is negligible in both risk groups, episodic risk has a strictly negative effect on transmissions. The effects of episodic risk on transmissions when the prevalence is *not* negligible (such as at the endemic equilibrium), are more complex, as seen in the next section.

### Endemic prevalence and the basic reproduction number as functions of the relative transmissibility during EHI and the re-selection rate

**Figure 1:**
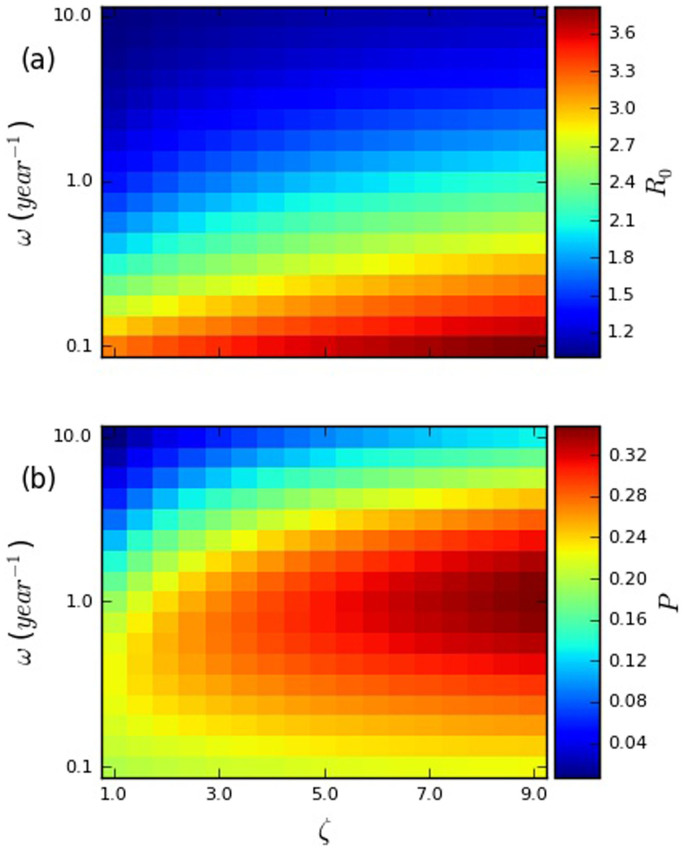
Heatmaps plotting (a) the basic reproduction number (*R*_0_), and (b) equilibrium prevalence (*P*) against the relative transmissibility during early HIV infection (*ζ*) and re-selection rate (*ω*). The per-act transmissibilities during EHI and chronic infection for each parameter set were chosen based on the constraints that (1) their ratio must be *ζ* and (2) the total transmission potential in a homogeneous system must be the same for all parameter sets. This transmission potential was chosen by setting the endemic prevalence for the lower-left parameter set to be 0.2. The other parameters used are summarized in Supplementary Table 1 in §2 of the Supplementary Information. Each panel has a scale that runs from the lowest to the highest value observed in that panel.

In Figure 1, we illustrate the effects of the relative transmissibility during EHI (*ζ*) and the re-selection rate (*ω*) on the basic reproduction number (*R*_0_) and the endemic prevalence (*P*), while fixing the total transmission potential across the course of infection, and keeping most other parameters the same (full details in §3.4 of the Supplementary Methods).

In Figure 1, both the basic reproduction number (*R*_0_) and the endemic prevalence (*P*) are monotonically increasing with respect to the relative transmissibility during EHI, even though the overall transmission potential is fixed. This is a consequence of the fact, noted above, that episodic risk reduces the boost in transmissions caused by risk heterogeneity less for transmissions during EHI than for transmissions during chronic infection. Consequently, if the total transmission potential is fixed, increasing transmissions from EHI at the expense of transmissions from chronic infection results in an increase in transmissions, leading to an increase in both *R*_0_ and *P*.

Unlike the relative transmissibility during EHI, the re-selection rate has a drastically different effect on the basic reproduction number and the endemic prevalence. As noted in the previous section, the basic reproduction number is monotonically decreasing with respect to the re-selection rate, because re-selection attenuates the increase in the average contact rate of infected individuals that results from risk heterogeneity. In contrast, the endemic prevalence has a non-monotonic dependence on the re-selection rate, peaking at a relatively high value of the latter. This is a product of the combined effects of the attenuation of the average contact rate of infected individuals (which works to reduce the endemic prevalence, just as it does the basic reproduction number) and the increased replenishment of higher risk susceptibles, which works to increase the endemic prevalence^6^. That episodic risk can increase endemic prevalence relative to the basic reproduction number at a given observed degree of (instantaneous) risk heterogeneity may explain the difficulty that modelers have experienced in reconciling estimates of the coefficient of variation in contact rate and the basic reproduction number with observed prevalence of HIV infection among MSM^10^.

### System determinants of key epidemiological measures

We now introduce a third outcome of interest: the effective treatment rate required to achieve elimination (*τ_E_*). As noted in the introduction, we collapse the treatment cascade into a single parameter, which we refer to as the *effective treatment rate* and denote *τ*. We then define the effective treatment rate required to achieve elimination (*τ_E_*) as the minimum value of the effective treatment rate (*τ*) at which elimination is (deterministically) achieved, i.e. the minimum value that reduces the basic reproduction number (*R*_0_) to or below the endemic threshold of *R*_0_ = 1. In practice, achieving elimination in a reasonable timeframe may require that *R*_0_ be brought significantly below 1. Consequently, if this model or one like it were to be used to set goals for a particular UT8T campaign, it would likely be desirable to target a lower basic reproduction number. Rather than fixing realistic targets, here we merely seek to provide qualitative insights.

**Figure 2:**
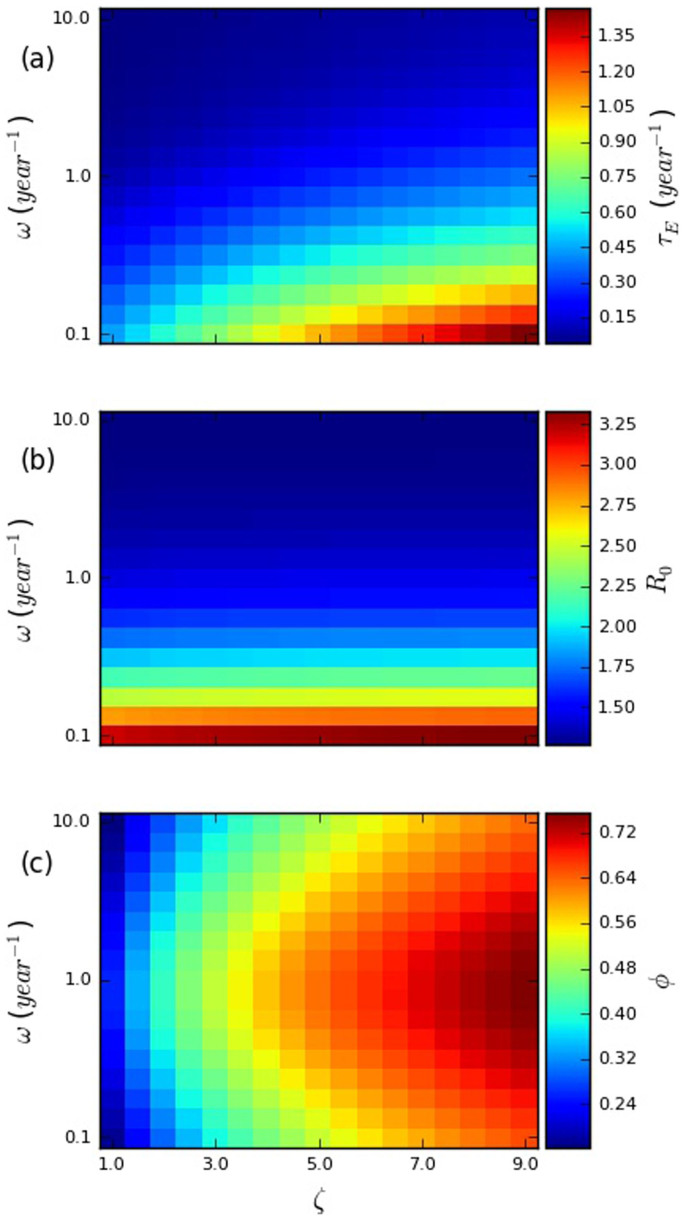
Heatmaps plotting (a) the effective treatment rate required to achieve elimination (*τ_E_*), (b) the basic reproduction number (*R*_0_), and (c) the fraction of transmissions from early HIV infection (*φ*) against the relative transmissibility during early HIV infection (*ζ*) and re-selection rate (*ω*). The other parameters used are summarized in Supplementary Table 1 in §2 of the Supplementary Information. Each panel has a scale that runs from the lowest to the highest value observed in that panel.

In Figure 2, we consider the effects on the basic reproduction number (*R*_0_), fraction of transmissions from early infection (*φ*), and effective treatment rate required to achieve elimination (*τ_E_*) of the same parameters examined in Figure 1, the relative transmissibility during early infection (*ζ*) and the re-selection rate (*ω*). In this case, however, instead of fixing the total transmission potential over the course of infection, we fix the endemic prevalence, at 0.2. We vary the total transmission potential (by varying *β*_1_ and *β*_2_, proportionally) in order to achieve this fixed endemic prevalence. This provides the common perspective in risk factor and prevention effects for HIV infection, where the prevalence is known, but what is determining that prevalence is unknown.

An important aspect of these relationships is that both factors have strong effects on the effective treatment rate required to achieve elimination as shown in panel (a) of Figure 2. Increasing the re-selection rate from 0.1 per year (relatively static risk groups) to 10.0 per year (strongly episodic risk) while holding the relative transmissibility during early infection constant at 9 results in a reduction of the required effective treatment rate from 1.47 to 0.101. This is over a 14-fold difference, without any change in the biological parameters, nor in any behavioral parameters that could be readily measured cross-sectionally. Likewise, reducing the relative transmissibility during early infection from 9 to 1 (while holding the re-selection rate constant at 0.1) results in a reduction of the necessary effective treatment rate to 0.442 – over a three-fold difference.

To understand what is generating these effects, it is useful to understand how the effective treatment rate required to achieve elimination (*τ_E_*) is affected by changes in the basic reproduction number (*R*_0_, shown as the outcome in panel (b) of Figure 2) and the fraction of transmissions from early infection (*φ*, shown as the outcome in panel (c) of Figure 2). As can be seen in Figure 2, the effect on *τ_E_* of the parameters *ω* (the re-selection rate) and *ζ* (the relative transmissibility during EHI) is essentially a combination of their effects on *R*_0_ and *φ*. This combination of effects is attributable to the fact that, as mentioned in the introduction, *R*_0_ and *φ* each represent a different aspect of difficulty in eliminating transmissions through UT8T: The higher *R*_0_ is, the larger the fraction of transmissions that must be prevented, and the higher *φ* is, the less effective UT8T is at preventing transmissions. A more detailed consideration of how *R*_0_ and *φ* affect *τ_E_* can be found in §4.1 of the Supplementary Results.

In panel (c) of Figure 2, we can see that the fraction of transmissions from EHI (*φ*) is, unsurprisingly, strongly positively dependent on the relative transmissibility during EHI (*ζ*). What is more striking is the strong, but non-monotonic, dependence on the rate of risk-group re-selection (*ω*), peaking when *ω* ≈ 1/year. The explanation for this phenomenon is that the fraction of transmissions from EHI is maximized when the difference in average contact rates between EHI and chronic infection is maximized, and this happens at a moderate re-selection rate: At very low re-selection rates, there is little difference, because the vast majority of infected individuals still have the same contact rate as when they were infected, regardless of their stage of infection. At very high re-selection rates, there is again little difference, because the vast majority of infected individuals have had their contact rate re-selected since they were infected, again, regardless of their stage of infection. It is only at intermediate re-selection rates that individuals in chronic infection are substantially more likely to have had their contact rates re-selected since being infected than individuals in EHI. Further details are included in §4.2 of the Supplementary Results.

### Endemic Prevalence as a function of *R*_0_

**Figure 3:**
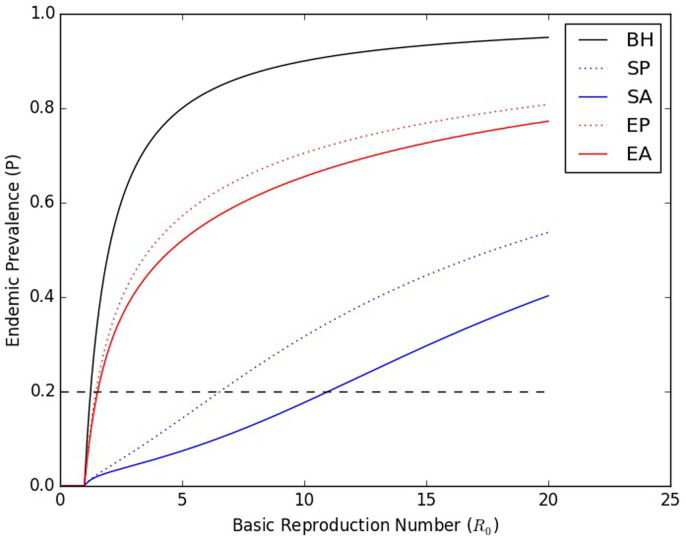
Curves showing the prevalence as a function of *R*_0_ when all parameters except the overall transmissibility are held constant. The curves shown are for the full (maximal) model (episodic risk, with assortative mixing (*m* = 0.5) – EA), and several reduced models: Episodic risk, with proportional mixing (EP, the primary model in this paper); Static risk heterogeneity, with assortative mixing (*m* = 0.5) (SA); Static risk heterogeneity, with proportional mixing (SP), and Behavioral Homogeneity (BH). The formulation of these reduced models is discussed in more detail in §3.5 of the Supplementary Methods. The dashed black line indicates a constant prevalence of 0.2, illustrating the drastically different values of *R*_0_ that are possible when the prevalence is fixed.

In order to illustrate the importance of various aspects of the model in determining the basic reproduction number *R*_0_, we plotted curves relating *R*_0_ to the endemic prevalence (*P*) for several related models. We derived a maximal model (parameter set given in Supplementary Table 1 in §2 of the Supplementary Information) from our primary model by adding assortative mixing (*m* = 0.5), in which individuals in a particular risk group are more likely to form contacts with other individuals in that risk group^8^,^11^, to our primary model. We derived static risk heterogeneity sub-models from both the maximal model and our primary model by setting the re-selection rate (*ω*) to 0. We further derived a behavioral homogeneity model by setting the ratio of the contact rates of the two risk groups (*r_HL_*) to 1. Further details are included in §3.5 of the Supplementary Methods. The results are shown in Figure 3.

**Figure 4:**
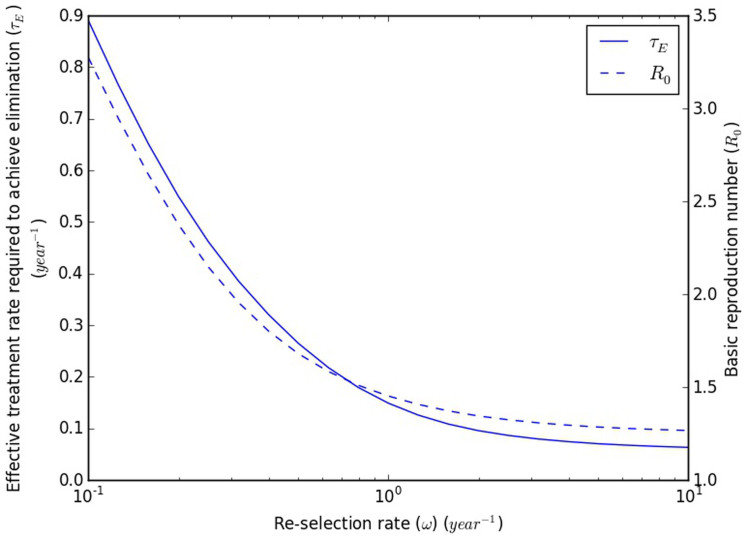
The basic reproduction number (*R*_0_) and effective treatment rate required to achieve elimination (*τ_E_*) as a function of the re-selection rate (*ω*). The prevalence is fixed at 0.2, and the fraction of transmissions from EHI (*φ*) is fixed at 0.447^14^. To achieve this, the transmissibilities from each of acute and chronic infection are allowed to vary; all other parameters are fixed. Details are presented in §3.7 of the Supplementary Methods.

It is particularly instructive to consider what effect transitioning between these submodels has when the prevalence is fixed, as it is in Figures 2 and 4. Because Figure 3 depicts prevalence as a function of *R*_0_, this amounts to selecting a horizontal line (such as the black dashed line, indicating a prevalence of 0.2), and examining how transitioning between curves changes the value of *R*_0_ at which the curve intersects that horizontal line. By doing so, we can see how drastically an estimated *R*_0_ can vary at a single prevalence, depending on the model used to estimate it. Failing to account for risk heterogeneity at all, or for assortative mixing, can result in a drastic underestimate of *R*_0_, while failing to account for episodic risk can result in a drastic *over*estimate. Such an error can easily result in incorrect inferences about the feasibility of achieving control or elimination with a given intervention.

Consistent with the discussion in the section *Endemic prevalence and the basic reproduction number as functions of the relative transmissibility during EHI and the re-selection rate*, static risk heterogeneity dramatically increases *R*_0_ at a given prevalence, but this increase is reduced (though not eliminated) when episodic risk is introduced. Assortative mixing increases the basic reproduction number at a given prevalence for both static risk heterogeneity and episodic risk, by causing the most frequently transmitting infected individuals (those in the higher risk group) to transmit preferentially to higher risk susceptibles.

It has previously been observed that static risk heterogeneity and assortative mixing both decrease prevalence at a given *R*_0_^12^,^13^. To this we now add that episodic risk increases prevalence at given *R*_0_, relative to static risk heterogeneity. In fact, all three of these observations reflect different manifestations of the same basic phenomenon: Provided that the dynamics of behavior and disease are independent (e.g. there is neither serosorting nor faster disease progression among certain behavioral groups), static risk heterogeneity and assortative mixing will decrease prevalence at a given *R*_0_ to the extent that they cause transmissions to disproportionately occur into a subgroup that saturates with infection more rapidly than the population as a whole. They do this by concentrating transmissions into the higher risk group, which consequently can become saturated with infection even when the prevalence in the population as a whole is still quite low. Episodic risk (partially) counteracts this effect (and thus increases prevalence at a given *R*_0_) by causing a net replacement of higher risk infecteds by (formerly) lower risk susceptibles, thereby reducing the saturation of the higher risk group, and by moving infected individuals into the lower risk group, where assortative mixing will increase the fraction of their contacts that are with susceptible individuals.

### Re-selection rate effects when the fraction of transmissions from EHI (*φ*) is fixed

In Figure 4, we illustrate the dramatic effects that the re-selection rate can have on the ease of elimination, even when the fraction of transmissions from early infection is fixed. In order to obtain the results in Figure 4, we fixed the endemic prevalence at 0.2 and the fraction of transmissions from early infection (*φ*) at 0.447, varying the per-contact transmission rate during chronic infection (*β*_2_) and the relative transmissibility during EHI (*ζ*) in order to do so. This value for phi was chosen based on the results of a recent phylodynamic analysis^14^. In this figure, changing the re-selection rate can be seen to result in *R*_0_ values that range from 1.27 to 3.28 and *τ_E_*s that range from 0.0635/year to 0.891/year.

To get a sense of the magnitude of the latter difference, let us suppose (1) that time from infection to diagnosis, time from diagnosis to linkage to care, and time from linkage to care to viral suppression are all exponentially distributed, and (2) that the effective treatment rate is the rate of (also exponentially distributed) single-step diagnosis, treatment, and viral suppression that generates a distribution of times from infection to suppression that has the same mean as the actual distribution. Using published estimates for the time from diagnosis to linkage to care (77.2% initiating care with 3 months)^15^ and the time from linkage to care to suppression (median of 1.03 years for all patients)^16^, we find that a *τ_E_* of 0.0635/year corresponds to a diagnosis rate of only 0.0710/year. In contrast, a *τ_E_* of 0.891/year would not be achievable at all with current times from linkage to care to viral suppression (median of 0.87 years even when restricted to patients with perfect retention in care)^16^, even if both diagnosis and linkage to care were instantaneous, and retention in care were perfect. Details of this calculation are presented in §4.3 of the Supplementary Results.

From these results, we can see that inferences about the feasibility of achieving control or elimination through UT8T are extremely dependent on assumptions about if and how individuals' contact rates change over the course of their lives, even when the prevalence, fraction of transmissions from EHI, and (instantaneous) distribution of contact rates are all known.

## Discussion

We have shown that episodic risk can have a dramatic effect on the effort required to achieve elimination through treatment, even when both the prevalence and the fraction of transmissions from early infection are fixed. Moreover, we have shown that episodic risk can have a major effect on the fraction of transmissions from early infection itself, independent of biological factors that increase transmission during EHI. We have also shown how differences in the extent to which risk heterogeneity is episodic can make the difference between a transmission system that is extremely vulnerable to universal test and treat, and one for which no level of testing and treating can ever be sufficient, by itself, to eliminate ongoing HIV transmission. Others have illustrated how transmission during early infection can affect the relationship between prevalence and the basic reproduction number, and thus alter the population effects of early diagnosis and treatment, sometimes in unexpected ways^4^,^5^. We have illustrated here how episodic risk is likely to considerably amplify those effects. Consequently, collecting more data on risk heterogeneity and episodic risk (or its continuous generalization, risk volatility^17^) is highly important in order to make the best possible decisions about how to allocate finite HIV control resources.

Our models do not attempt to reproduce real-world transmission dynamics. They dichotomize what are actually continuous phenomena: transmissibility over the course of untreated infection, contact rates, change in contact rates, and treatment success or failure. They are kept simple in order to focus on the basic concepts of how changes in contact rates over the course of individuals' lives alter the fraction of transmissions from early infection, *R*_0_, and the effectiveness of UT8T. This understanding should affect how control decisions are pursued in the face of ignorance about how individuals change their contact rates over the course of their lives. It should also affect both field studies and modeling studies designed to reduce our ignorance. The simplicity of our models, however, mean that any attempt to predict the effectiveness of UT8T in a particular population based on our results is invalid in the absence of an assessment of how realistic relaxation of our simplifying assumptions affects our results. However, we have identified important qualitative phenomena which will be highly relevant even in more realistic models.

In this paper, we have presented episodic risk abstractly, without considering its determinants. As noted in a previous paper^6^, there are numerous causal phenomena that can result in periods of more frequent or riskier sexual activity than characterizes an individual's sexually active life as a whole, such as periods of recreational drug use or alternation between a partnered state (with a low or zero rate of outside sexual contacts) and an unpartnered state (with a higher rate of casual sex). Higher rates of sexual activity at certain ages can also produce similar phenomena, particularly when sexual activity shows assortative mixing by age, as is generally the case. Some of these effects have been captured in previous model analyses that have explored the role of EHI on transmission in populations^18^,^19^. But empirical work on these factors has not progressed. We hope that our simple way of abstracting risk behavior fluctuations with our episodic risk formulation helps to show why risk fluctuations are so important. But we are not advocating that the episodic risk abstraction should be the way that histories are obtained. How risk fluctuation histories are taken should depend on what questions are easiest to comprehend and validly answer and how well the answers to those questions allow researchers to measure changes in risk behavior over the course of an individual's life.

One consistent pattern that we have observed across various parameters that we have examined is that changes to parameters tend to make elimination through UT8T harder when they create or strengthen the phenomenon of a distinct core group that transmits heavily to itself. That this phenomenon is consistent across various ways of strengthening such a group (increasing contact rate heterogeneity, decreasing episodic risk, and increasing assortativity) suggests that it is likely to be robust to realistic relaxation of simplifying assumptions. Under these conditions, UT8T becomes less effective. These conditions, however, form a core group with persistent higher risk behavior where Pre-Exposure Prophylaxis is more practicable and effective.

One possible direction for further research is extending the various dichotomized aspects of the model to polychotomous or continuous measures. For example, dichotomized episodic risk can be replaced by continuous risk volatility^17^,^20^. Another is incorporating important aspects of sexual behavior that are not included in the present model, such as long-term monogamous or semi-monogamous partnerships, temporal changes in insertive or receptive behavior^21^ or temporal changes in condom use with different types of partners.

Episodic risk behavior/risk volatility has been understudied. Two studies have found both between and within-individual volatility^22^,^23^. However, due to limitations in the datasets that were available, these were based on an analysis of data from the early 1990s, and therefore may not be reflective of current behavioral dynamics.

There are three fundamental messages that we hope that other researchers will take from this paper. First, we have presented a new methodology for calculating the basic reproduction number in the presence of episodic risk, which generalizes naturally to continuous risk volatility, and which helps in creating understanding not only of the reasons for the effects of episodic risk itself, but for its interactions with other behavioral or biological phenomena. We hope that this approach will be of service to other modelers. Second, we have shown how episodic risk can increase prevalence at the same time as it reduces the basic reproduction number and the difficulty of eradication. Third, we have shown how strong these effects can be. Consequently, they should be taken into account, and the necessary data gathered in order to determine their magnitude and better plan control activities using models that incorporate the strong effects we have illustrated.

## Methods

Throughout this paper, all sexual behavior is modeled as instantaneous, symmetric contacts. All sexual contacts between an infected individual and a susceptible individual are assumed to have a potential for HIV transmission that depends only on the stage and treatment status of the infected individual. Per-contact transmissibility is in general higher during EHI than during chronic infection, with the ratio between the two transmissibilities being a parameter whose effects we explore.

**Figure 5:**
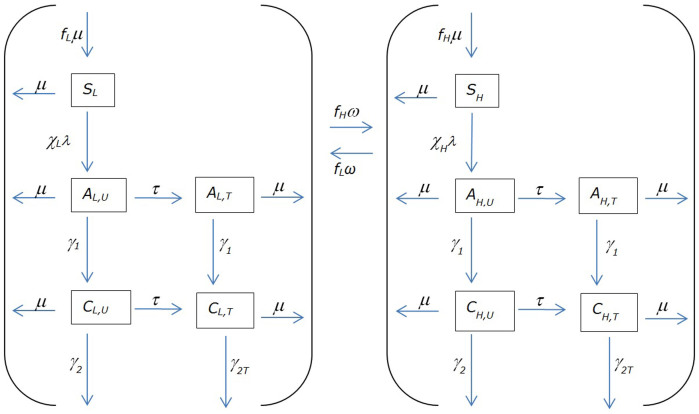
Dynamics of acquisition, progression, and treatment of infection. The meanings of symbols representing subpopulations are given in Table 1, and the meanings of symbols representing parameters (including derived parameters) are given in Table 2. Note that only re-selections resulting in transition to the other risk group are shown; loops depicting re-selections that place the individual in the same risk group (and therefore same compartment) they were in prior to re-selection are not included.

The flow diagram for our primary model is depicted in Figure 5. More details, parameter definitions and ranges, and a system of coupled differential equations are presented in §3.1 of the Supplementary Methods.

All simulations were performed and numerical results obtained using the Anaconda distribution (Continuum Analytics, version 2.1.0, 64-bit) of Python (version 3.4.1). Apart from the standard library, we made use of the NumPy (version 1.9.0) and SciPy (version 0.14.0) packages for simulation and computation, and the matplotlib (1.4.0) package for visualization and graphics production. All integration of systems of ordinary differential equations (ODEs) was done using the scipy.integrate.odeint function, with default solver and stepsize. Further details may be found in the source code, which is attached at the end of the Supplementary Information.

In general, we have three outcomes of interest: the basic reproduction number (*R*_0_), the fraction of transmissions from EHI (*φ*), and the effective treatment rate that is necessary to completely eliminate ongoing transmission (*τ_E_*). For the purposes of this paper, we use the next-generation matrix (NGM) definition of *R*_0_ developed by Diekmann et al.^1^ We define *φ* as the fraction of transmissions from EHI made by all individuals in a generation during exponential growth (under the same assumptions used to define the next-generation matrix), and solve for it using a modification of the methods for finding *R*_0_^1^, discussed in §3.6 of the Supplementary Methods. Using these definitions, we obtain an algebraic equation giving *τ_E_* in terms of *R*_0_ and *φ* for sub-models without episodic risk. This equation is presented, along with its implications, in §3.6 of the Supplementary Methods.

Except where otherwise noted, the endemic prevalence in the absence of treatment was set to equal 0.2, and for each parameter set, a value of *β*_2_ that generated that endemic prevalence was obtained numerically. All of the software listed above is free to use. Source code is included as §5 of the Supplementary Information.

## Supplementary Material

Supplementary Information Supplementary Information

## Figures and Tables

**Table 1:** Symbols representing (sub) populations

Symbol	Meaning
*L*	Total population of lower risk individuals
*S_L_*	Susceptible, lower risk
*A_L,U_*	Early-infected, lower risk, untreated
*A_L,T_*	Early-infected, lower risk, treated
*C_L,U_*	Chronically infected, lower risk, untreated
*C_L,T_*	Chronically infected, lower risk, treated
*H*	Total population of higher risk individuals
*S_H_*	Susceptible, higher risk
*A_H,U_*	Early-infected, higher risk, untreated
*A_H,T_*	Early-infected, higher risk, treated
*C_H,U_*	Chronically infected, higher risk, untreated
*C_H,T_*	Chronically infected, higher risk, treated

**Table 2:** Symbols representing parameters (including derived parameters)

Symbol	Unit	Value(s)	Meaning
*χ*	1/year	20	Average contact rate
*γ*_1_	1/year	1	Rate of transition from EHI to chronic infection
*γ*_2_	1/year	1/8.45	Rate of AIDS-related death or departure from the sexually active population during chronic infection, if untreated. Chosen to give a mean total duration of infection of 9.45 years^24^.
*γ*_2*T*_	1/year	0	Rate of AIDS-related death or departure from the sexually active population during chronic infection, if treated. Does not actually affect any results presented in this paper.
*μ*	1/year	1/40.28	Rate of death or departure from the sexually active population unrelated to HIV infection; because the population size is normed to be 1 at the disease-free equilibrium, this is also the absolute rate of entry into the sexually active population. From Supplementary Text S3 of Volz et al.^14^.
*τ*	1/year	Variable	Effective treatment rate
*r_HL_*	-	Variable	Contact rate ratio between higher risk and lower risk individuals
*f_H_*	-	Variable	Fraction of the population that is at higher risk at the disease-free equilibrium.
*f_L_*	-	1 − *f_H_*	Fraction of the population that is at lower risk at the disease-free equilibrium.
*χ_L_*	1/year	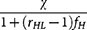	Contact rate for lower risk individuals
*χ_H_*	1/year	*r_HL_χ_L_*	Contact rate for higher risk individuals
*m*	-	Variable (0–1.0)	Fraction of an individual's contacts that are reserved for members of the same risk group. Throughout the main text, it is 0 unless stated otherwise.
*ω*	1/year	Variable (0.01–10.0)	Rate at which we randomly re-select which risk group an individual is in (re-selection rate)
*ω_H_*	1/year	*f_L_ω*	Rate at which higher risk individuals transition to the lower risk group
*ω_L_*	1/year	*f_H_ω*	Rate at which lower risk individuals transition to the higher risk group
*ζ*	-	Variable (1.0–8.0)	Relative transmissibility during EHI
*β*_1_	transmissions/contact	*ζβ*_2_	Per-contact transmissibility during EHI
*β*_2_	transmissions/contact	[used to fit the model to a target prevalence or basic reproduction number]	Per-contact transmissibility during chronic infection
*H*_1_	transmissions	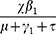	The average number of secondary transmissions during EHI per index case, under behavioral homogeneity.
*H*_2_	transmissions	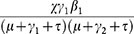	The average number of secondary transmissions during chronic infection per index case, under behavioral homogeneity.
*H*	transmissions	*H*_1_ + *H*_2_	The average number of secondary transmissions, under behavioral homogeneity.
*ψ*_1_		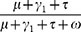	The average fraction of time spent in untreated EHI that occurs prior to the first re-selection of that individual's contact rate after his infection.
*ψ*_2_		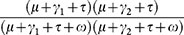	The average fraction of time spent in untreated chronic infection that occurs prior to the first re-selection of that individual's contact rate after his infection.
*ψ*		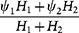	The average fraction of secondary transmissions (under behavioral homogeneity) that occur prior to the first re-selection of that individual's contact rate after his infection.
