# Supplementary material for: Strong influence of behavioral dynamics on the ability of testing and treating HIV to stop transmission
Source: Sci Rep. 2015 Apr 22;5:9467. doi: 10.1038/srep09467 (PMC5386110; doi:10.1038/srep09467)
Supplement: Supplementary Information [file srep09467-s1.doc]

Strong influence of behavioral dynamics on the ability of testing and treating HIV to stop transmission

Christopher J. Henry^1*^ & James S. Koopman^1^

^1^University of Michigan

Supplementary Information

§1 Supplementary Figures


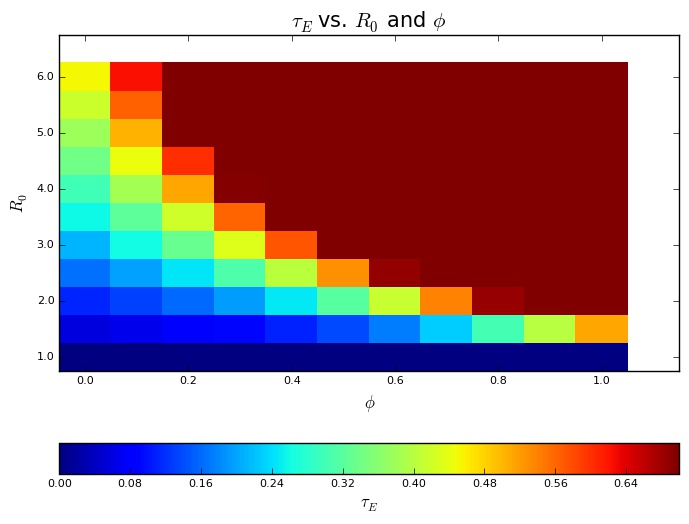


**Supplementary Figure 1.** Effective treatment rate required to achieve elimination of ongoing transmission (*τ_E_*) vs. basic reproduction number (*R*_0_) and fraction of transmissions from early infection (*φ*), under behavioral homogeneity. All values are 0 when *R*_0_ = 1; and the maximum value obtained (when R_0_ = 6 and *φ* = 1) is 5.12.


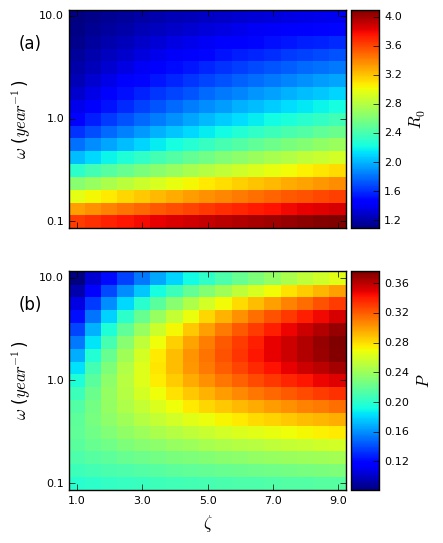


**Supplementary Figure 2.** Heatmaps plotting (a) the basic reproduction number (*R*_0_), and (b) equilibrium prevalence (*P*) against the relative transmissibility during early HIV infection (*ζ*) and re-selection rate (*ω*), in the elaborated model. The average per-contact transmissibilities during EHI and chronic infection for each parameter set were chosen based on the constraints that (1) their ratio must be *ζ* and (2) the total transmission potential in a homogeneous system must be the same for all parameter sets. This transmission potential was chosen by setting the endemic prevalence for the lower-left parameter set to be 0.2. The other parameters used are summarized in *Supplementary Table 1*. Each panel has a scale that runs from the lowest to the highest value observed in that panel.


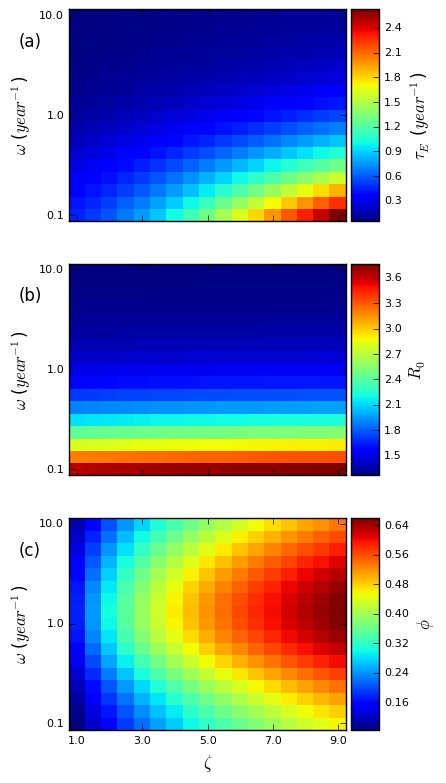


**Supplementary Figure 3.** Heatmaps plotting (a) the effective treatment rate required to achieve elimination (*τ_E_*), (b) the basic reproduction number (*R*_0_), and (c) the fraction of transmissions from early HIV infection (*φ*) against the relative transmissibility during early HIV infection (*ζ*) and re-selection rate (*ω*), in the elaborated system. The other parameters used are summarized in *Supplementary Table 1*. Each panel has a scale that runs from the lowest to the highest value observed in that panel.


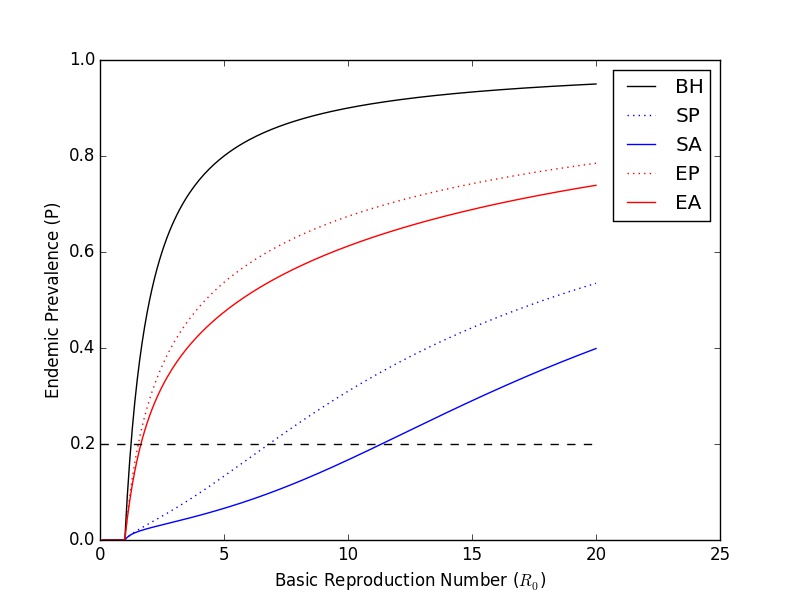


**Supplementary Figure 4.** Curves showing the prevalence as a function of *R*_0_ when all parameters except the overall transmissibility are held constant, in the elaborate system. The curves shown are for the full (maximal) model (episodic risk, with assortative mixing – EA), and several reduced models: Episodic risk, with proportional mixing (EP, the primary model in this paper); Static risk heterogeneity, with assortative mixing (SA); Static risk heterogeneity, with proportional mixing (SP), and Behavioral Homogeneity (BH). The formulation of these reduced models is discussed in more detail in *Supplementary Methods: Construction of submodels for Figure 3*. The dashed black line indicates a constant prevalence of 0.2, illustrating the drastically different values of *R*_0_ that are possible when the prevalence is fixed.


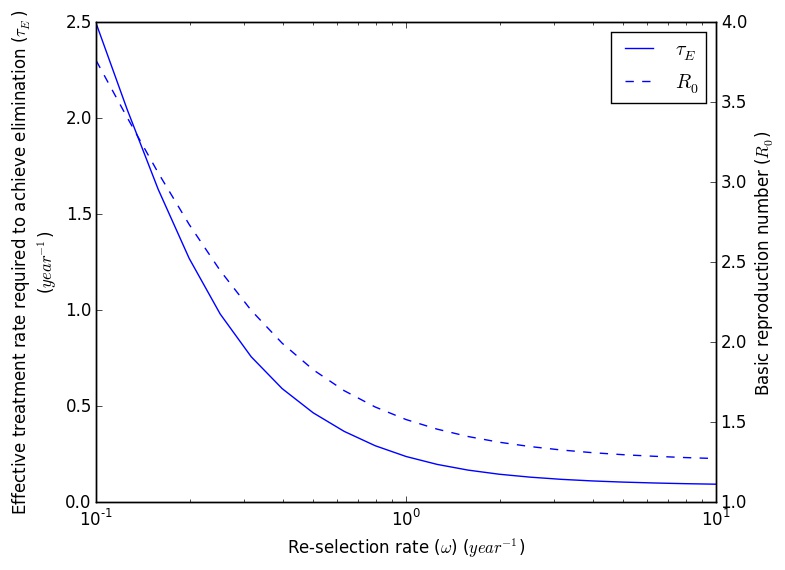


**Supplementary Figure 5.** The basic reproduction number (*R*_0_) and effective treatment rate required to achieve elimination (*τ_E_*) as a function of the re-selection rate (*ω*). The prevalence is fixed at 0.2, and the fraction of transmissions from EHI (*φ*) is fixed at 0.447^[6]^. To achieve this, the transmissibilities from each of acute and chronic infection are allowed to vary; all other parameters are fixed. Details are presented in *Supplementary Methods: Construction of Figure 4*.


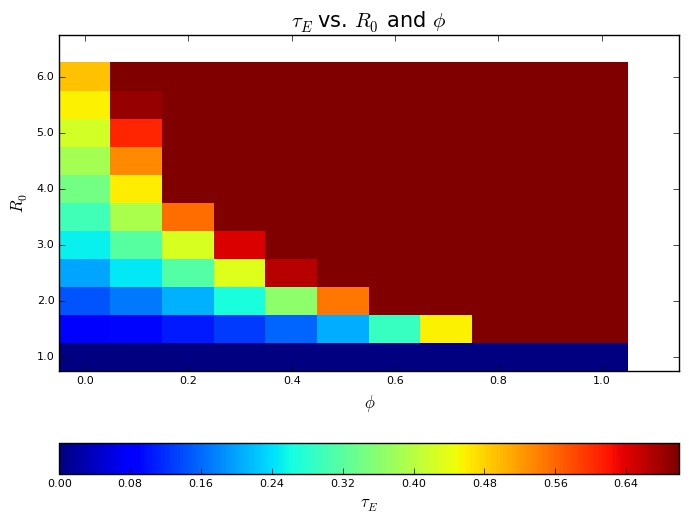


**Supplementary Figure 6.** Effective treatment rate required to achieve elimination of ongoing transmission (*τ_E_*) vs. basic reproduction number (*R*_0_) and fraction of transmissions from early infection (*φ*), under behavioral homogeneity, in the elaborated model. All values are 0 when *R*_0_ = 1; and the maximum value obtained (when R_0_ = 6 and *φ* = 1) is 14.8.

§2 Supplementary Tables

**Table 1.** Default parameters. All figures and results in this paper use the following parameter set, except as explicitly noted. Only parameters with “variable” values in Table 1 of the main text are shown here.

| Symbol | Unit | Value(s) | Meaning |
| --- | --- | --- | --- |
| *r_HL_* | - | 21 | Contact rate ratio between higher risk and lower risk individuals. |
| *f_H_* | - | 0.1 | Fraction of the population that is in the higher risk group at the disease-free equilibrium. |
| *m* | - | 0 | Fraction of an individual’s contacts that are reserved for members of the same risk group, i.e. the assortativity coefficient^1^. |
| *ω* | 1/year | 1 | Rate at which we randomly re-select which risk group an individual is in (re-selection rate) |
| *ζ* | - | 10 | Relative transmissibility during EHI. |
| *τ* | 1/year | 0 | Effective treatment rate. |

**Table 2.** Derived parameters used in the calculation and presentation of *R*_0_.

| Symbol | Value | Meaning |
| --- | --- | --- |
| *ν*_1_ | $\mu+\gamma_{1}+\tau$ | Total rate of leaving the early-infected and untreated state. |
| *ν*_1_ | $\mu+\gamma_{2}+\tau$ | Total rate of leaving the chronically infected and untreated state. |
| *g_H_* | $\frac{f_{H}r_{HL}}{1+f_{H}\left(r_{HL}-1\right)}$ | Fraction of all contacts that are made by higher risk individuals at the disease-free equilibrium. |
| *g_L_* | $\frac{f_{L}}{1+f_{H}\left(r_{HL}-1\right)}$ | Fraction of all contacts that are made by lower risk individuals at the disease-free equilibrium. |
| *c_V_*(*χ*)^2^ | $\frac{f_{H}f_{L}\left(r_{HL}-1\right)^{2}}{\left(1+f_{H}\left(r_{HL}-1\right)\right)^{2}}$ | The variance of the contact rate divided by the square of the mean contact rate (i.e. the coefficient of variation squared). |
| *r* | $\frac{mr_{HL}}{\left(1+f_{H}\left(r_{HL}-1\right)\right)^{2}}$ | Scaled assortativity, used in calculating R_0_. |
| *H*_1_ | $\frac{\chi\beta_{1}}{\nu_{1}}$ | The contribution of transmissions during EHI to *R*_0_ in the corresponding behaviorally homogeneous system. |
| *H*_2_ | $\frac{\chi\gamma_{1}\beta_{2}}{\nu_{1}\nu_{2}}$ | The contribution of transmissions during chronic infection to *R*_0_ in the corresponding behaviorally homogeneous system. |
| *ψ*_1_ | $\frac{\nu_{1}}{\nu_{1}+\omega}$ | The fraction of the average time spent in untreated EHI that occurs prior to the first re-selection of that individual’s contact rate after his infection. |
| *ψ*_2_ | $\frac{\nu_{1}\nu_{2}}{\left(\nu_{1}+\omega\right)\left(\nu_{2}+\omega\right)}$ | The fraction of the average time spent in untreated chronic infection that occurs prior to the first re-selection of that individual’s contact rate after his infection. |
| *H* | $H_{1}+H_{2}$ | The contribution of all transmissions to *R*_0_ in the corresponding behaviorally homogeneous system (i.e. *R*_0_ for the corresponding behaviorally homogeneous system). |
| *ψ* | $\frac{\psi_{1}H_{1}+\psi_{2}H_{2}}{H_{1}+H_{2}}$ | The fraction of the average number of secondary transmissions per index case, during exponential growth and under behavioral homogeneity, that occur prior to the first re-selection of that individual’s contact rate after his infection. |

Table 3. Stage of infection parameters for the elaborated model.

| Stage number | Grouped under | Mean Duration (1/*γ_i_*) | Per-contact transmission rate (*β_i_*) |
| --- | --- | --- | --- |
| 1 | Acute | 1 week | 0.003 |
| 2 | Acute | 1 week | 0.03 |
| 3 | Acute | 1 week | 0.04 |
| 4 | Acute | 1 week | 0.03 |
| 5 | Acute | 16.6 weeks | 0.02 |
| 6 | Chronic | 1.9 years | 0.0007 |
| 7 | Chronic | 1.9 years | 0.0007 |
| 8 | Chronic | 1.9 years | 0.0007 |
| 9 | Chronic (“Early AIDS”) | 0.9 years | 0.006 |

§3 Supplementary Methods

§3.1 Detailed description of the underlying transmission model

Our model of transmission dynamics is a deterministic compartmental model (DCM), but, as discussed in the main text, we will frequently refer to “individuals” to describe the meaning of various flows, using the fact that a DCM is the limiting case of an individual-based model in an infinitely large, thoroughly-mixing population. For convenience, the total population size is set equal to 1 at the disease free equilibrium. Consequently, the “birth” (entry into the population of sexually active MSM) rate is equal to the natural “mortality” (departure from the sexually active population) rate *μ*. For simplicity, we assume that this birth rate does not depend on the size of the current population of MSM. Throughout this paper, *μ* is fixed at .0248 / year. There is no age structure in our model. We also exclude importation of HIV from outside the population from our model.

Individuals are divided into two risk groups, differentiated by their (sexual) contact rates, which we denote *χ_H_* for the higher risk group and *χ_L_* for the lower risk group. We parameterize this division by the fraction of individuals who enter the population in the higher risk group (*f_H_*), and the ratio of contact rates between the two groups (*r_HL_*). The mean contact rate *χ* is fixed at 20 / year. All contacts are assumed to be instantaneous and symmetric. Individuals in each risk group reserve a fraction *m* of their contacts for members of their own risk group (0 by default), with all other contacts being made proportionately.

Individuals do not necessarily remain in the same risk group for the full duration of their stay in the sexually active population; we re-select which risk group they are in at a rate *ω*, which we refer to as the “re-selection rate.” When we do so, we assign them to each risk group with the same probability as when they first entered the sexually active population; consequently, *f_H_* is also the fraction of individuals who are in the higher risk group at the disease-free equilibrium.

We simplify the natural history of infection to a relatively brief early phase (early HIV infection, EHI) followed by a longer chronic phase. During early infection, the per-act transmission probability is *β*_1_, and during chronic infections, it falls to *β*_2_. *β*_2_ is in general allowed to vary in order to attain a desired endemic prevalence or basic reproduction number; *β*_1_ is the product of *β*_2_ and the relative transmissibility during EHI (relative to chronic infection), which we denote *ζ*. Progression from early to chronic infection occurs at a rate of *γ*_1_ (1 / year throughout this paper), regardless of treatment status, and “progression” from chronic infection to death or departure from the sexually active population due to HIV infection occurs at a rate of *γ*_2_ (1/8.45 years) if untreated, and does not occur if treated ($\gamma_{2T}=0$) – note, however, that none of the calculations or results in this paper depend on the value of *γ*_2_*_T_*.

The treatment cascade is simplified to a binary distinction between individuals who are “effectively treated” (those who have attained sustained viral suppression) and individuals who are not (everyone else). Transition from the latter group to the former occurs at an “effective treatment rate” of *τ*. To simplify the model, we do not include virologic failure after viral suppression, and we assume that virally suppressed individuals are completely non-transmitting.

The above-described dynamics are summarized in the following set of linked differential equations (symbols denoting subpopulations are defined in Table 1 of the main text):

$$\begin{matrix} \frac{d}{dt}\left(S_{L}\right)=f_{L}\mu-\left(\mu+\chi_{L}\lambda_{L}+f_{H}\omega\right)S_{L}+f_{L}\omega S_{H} \\ \frac{d}{dt}\left(S_{H}\right)=f_{H}\mu-\left(\mu+\chi_{H}\lambda_{H}+f_{L}\omega\right)S_{H}+f_{H}\omega S_{L} \\ \frac{d}{dt}\left(A_{L,U}\right)=\chi_{L}\lambda_{L}S_{L}-\left(\mu+\gamma_{1}+f_{H}\omega+\tau\right)A_{L,U}+f_{L}\omega A_{H,U} \\ \frac{d}{dt}\left(A_{H,U}\right)=\chi_{H}\lambda_{H}S_{H}-\left(\mu+\gamma_{1}+f_{L}\omega+\tau\right)A_{H,U}+f_{H}\omega A_{L,U} \\ \frac{d}{dt}\left(C_{L,U}\right)=\gamma_{1}A_{L,U}-\left(\mu+\gamma_{2}+f_{H}\omega+\tau\right)C_{L,U}+f_{L}\omega C_{H,U} \\ \frac{d}{dt}\left(C_{H,U}\right)=\gamma_{1}A_{H,U}-\left(\mu+\gamma_{2}+f_{L}\omega+\tau\right)C_{H,U}+f_{H}\omega C_{L,U} \\ \frac{d}{dt}\left(A_{L,T}\right)=\tau A_{L,U}-\left(\mu+\gamma_{1}+f_{H}\omega\right)A_{L,T}+f_{L}\omega A_{H,T} \\ \frac{d}{dt}\left(A_{H,T}\right)=\tau A_{H,U}-\left(\mu+\gamma_{1}+f_{L}\omega\right)A_{H,T}+f_{H}\omega A_{L,T} \\ \frac{d}{dt}\left(C_{L,T}\right)=\tau C_{L,U}+\gamma_{1}A_{L,T}-\left(\mu+\gamma_{2T}+f_{H}\omega\right)C_{L,T}+f_{L}\omega C_{H,T} \\ \frac{d}{dt}\left(C_{H,T}\right)=\tau C_{H,U}+\gamma_{1}A_{H,T}-\left(\mu+\gamma_{2T}+f_{L}\omega\right)C_{H,T}+f_{H}\omega C_{L,T} \\ \text{where} \\ \lambda_{L}=m\left(\frac{\beta_{1}A_{L,U}+\beta_{2}C_{L,U}}{L}\right)+\left(1-m\right)\left(\frac{\beta_{1}\left(\chi_{L}A_{L,U}+\chi_{H}A_{H,U}\right)+\beta_{2}\left(\chi_{L}C_{L,U}+\chi_{H}C_{H,U}\right)}{\chi_{L}L+\chi_{H}H}\right) \\ \lambda_{H}=m\left(\frac{\beta_{1}A_{H,U}+\beta_{2}C_{H,U}}{H}\right)+\left(1-m\right)\left(\frac{\beta_{1}\left(\chi_{L}A_{L,U}+\chi_{H}A_{H,U}\right)+\beta_{2}\left(\chi_{L}C_{L,U}+\chi_{H}C_{H,U}\right)}{\chi_{L}L+\chi_{H}H}\right) \end{matrix}$$

§3.2 Calculation of transmission potentials

The rate of leaving untreated EHI is simply the sum of the rates of dying, progressing to chronic infection, and becoming treated: $\nu_{1}=\mu+\gamma_{1}+\tau$. Therefore, the average time spent in untreated EHI is $\frac{1}{\nu_{1}}$. During exponential growth, and under behavioral homogeneity, the transmission rate during this time is $\chi\beta_{1}$. Consequently, the expected number of transmissions during EHI per index case (the transmission potential from EHI) is

$H_{1}=\frac{\chi\beta_{1}}{\nu_{1}}$.

Similarly, the expected number of transmissions during chronic infection per index case that reaches chronic infection untreated is $\frac{\chi\beta_{2}}{\nu_{2}}$, where $\nu_{2}=\mu+\gamma_{2}+\tau$. However, only a fraction $\frac{\gamma_{1}}{\nu_{1}}$ of index cases reach chronic infection before dying or becoming treated. Consequently, the expected number of transmissions during chronic infection per index case (the transmission potential from chronic infection) is

$H_{2}=\frac{\chi\gamma_{1}\beta_{2}}{\nu_{1}\nu_{2}}$.

The total transmission potential is simply the sum of the transmission potentials from EHI and from chronic infection:

$H=H_{1}+H_{2}$.

§3.3 Calculation of remaining heterogeneity effects

Consider the subpopulation of individuals who are acutely infected and untreated, and whose contact rate has not been re-selected since they became infected. Individuals leave this subpopulation at a rate of *ν*_1_, and we re-select their contact rates at a rate of *ω*. Consequently, the fraction of acutely infected, untreated individuals whose contact rates we have not re-selected since their infection when they cease to be both acutely infected and untreated is

$\psi_{1}=\frac{\nu_{1}}{\nu_{1}+\omega}$.

Because the dynamics of this system are all first order, this is also the (average) fraction of time spent acutely infected and untreated that occurs prior to the first post-infection re-selection of an individual’s contact rate. Similarly, the fraction of time spent chronically infected and untreated *by individuals who enter chronic infection prior to the first post-infection re-selection of their contact rates* that occurs prior to the first post-infection re-selection of their contact rates is $\frac{\nu_{2}}{\nu_{2}+\omega}$. However, only *ψ*_1_ of individuals entering chronic infection untreated do so without having had their contact rates re-selected. Consequently, the fraction of time spent chronically infected and untreated by all individuals that occurs prior to the first post-infection re-selection of their contact rates

$\psi_{2}=\frac{\nu_{1}\nu_{2}}{\left(\nu_{1}+\omega\right)\left(\nu_{2}+\omega\right)}$.

Because *ψ* represents the fraction of secondary transmissions (during exponential growth and under behavioral homogeneity) that occur prior to the first post-infection re-selection of the transmitter’s contact rate, its numerator is the sum of the number of such transmissions during EHI and the number of such transmission during chronic infection, while its denominator is the sum of all transmissions during EHI and all transmissions during chronic infection:

$\psi=\frac{\psi_{1}H_{1}+\psi_{2}H_{2}}{H_{1}+H_{2}}$.

§3.4 Construction of Figure 1

We used the base parameters detailed in Supplementary Table 1, except for *ζ* and *ω*, which we varied, with ranges of 1-9 and 0.1-10 (the latter on a logarithmic scale), respectively. To begin, we found by numerical simulation values for *β*_1_ and *β*_2_ (related by the equation$\beta_{1}=\zeta\beta_{2}$) that would result in an endemic prevalence of 0.2 when *ζ* = 1 and *ω* = 0.1, corresponding to the lower left corner of each panel of Figure 1. We then calculated the total transmission potential *H*, using the formulas in Supplementary Table 2. For each of the remaining parameter sets, we began by setting *ζ* and *ω* for that parameter set, leaving the other parameters unchanged, and calculating the new total transmission potential *H*’. Because the total transmission potential is proportional to *β*_2_ when all other parameters are fixed (including *ζ*, but not including the derived parameter *β*_1_), and we wanted all parameter sets to have the same total transmission potential, we then multiplied *β*_2_ by the ratio $\frac{H}{H'}$.

For each of the parameter sets we found in this fashion, we found the endemic prevalence by numerical simulation and calculated *R*_0_ using the methods described in the *Calculation of outcomes given a parameter set* subsection of this Supplementary Methods section. These results were then plotted as heatmaps using the python function matplotlib.pyplot.pcolor. All code used is included at the end of the Supplementary Information.

§3.5 Construction of submodels for Figure 3

The standard model (episodic risk, proportional mixing; EP) uses the full parameter set given in Supplementary Table 1. For all of the other models, one or more parameters are changed: *ω*, indicating the degree to which risk heterogeneity is episodic, is set to 0 for the models SA (static risk heterogeneity, assortative mixing), SP (static risk heterogeneity, proportional mixing), and BH (behavioral homogeneity). *m*, indicating the degree to which mixing is assortative, is set to 0.5 in the models EA (episodic risk, assortative mixing) and SA. *r_HL_*, indicating the degree of risk heterogeneity, is set to 0 in model H only. Although model BH still technically contains two behavioral groups, they are now indistinguishable from each other in all respects, and are therefore effectively the same group.

§3.6 Calculation of outcomes given a parameter set

Our method for finding the basic reproduction number *R*_0_ is that developed by Diekmann et al.^2^ We will briefly review that method in the course of describing our use of it.

Given a set of differential equations for a transmission system, it is possible to construct a Jacobian matrix *J* for the linearization of the subsystem of infectious subpopulations around the disease-free equilibrium. This matrix can then be decomposed into the sum of a transmission matrix *T* and a transition matrix *Σ*. To do so for our model is straightforward, but produces relatively unwieldy equations; consequently, we will make use of several new symbols that we present in Supplementary Table 2. These symbols allow both our intermediate equations and our final expression for *R*_0_ to be more succinct; in the case of the latter, they also provide an increase in clarity.

In the following matrices, the order of compartments is (A_H,U_, A_L,U_, C_H,U_, C_L,U_); the remaining compartments represent non-infectious individuals, and are not part of the linearized system near the disease-free equilibrium. The transmission and transition matrices, for the system described above, are respectively:

$\begin{matrix} T=\left[\begin{matrix} \chi_{H}\beta_{1}\left(g_{H}+mg_{L}\right) & \chi_{L}\beta_{1}\left(1-m\right)g_{H} & \chi_{H}\beta_{2}\left(g_{H}+mg_{L}\right) & \chi_{L}\beta_{2}\left(1-m\right)g_{H} \\ \chi_{H}\beta_{1}\left(1-m\right)g_{L} & \chi_{L}\beta_{1}\left(g_{L}+mg_{H}\right) & \chi_{H}\beta_{2}\left(1-m\right)g_{L} & \chi_{L}\beta_{2}\left(g_{L}+mg_{H}\right) \\ 0 & 0 & 0 & 0 \\ 0 & 0 & 0 & 0 \end{matrix}\right] \\ \Sigma=\left[\begin{matrix} -\left(\nu_{1}+f_{L}\omega\right) & f_{H}\omega& 0 & 0 \\ f_{L}\omega& -\left(\nu_{1}+f_{H}\omega\right) & 0 & 0 \\ \gamma_{1} & 0 & -\left(\nu_{2}+f_{L}\omega\right) & f_{H}\omega\\ 0 & \gamma_{1} & f_{L}\omega& -\left(\nu_{2}+f_{H}\omega\right) \end{matrix}\right] \end{matrix}$.

Given these matrices, the basic reproduction number *R*_0_ can be defined as the dominant eigenvalue of the Next-Generation Matrix with Large Domain:

$K_{L}=-T\Sigma^{-1}$.

Diekmann et al.^2^ further note that the dominant eigenvalue of *K_L_* is the same as the dominant eigenvalue of the Next-Generation Matrix *K*, which is the submatrix of *K_L_* that contains only the rows and columns corresponding to states that individuals can have immediately upon infection (i.e., in this model, *A_L,U_* and *A_H,U_*). After some algebra, the Next-Generation Matrix can be shown to be:

$K=H\left(\begin{matrix} g_{H}+\left(\frac{r_{HL}}{r_{HL}-1}c_{V}\left(\chi\right)^{2}+f_{L}r\right)\psi& g_{H}+\left(-\frac{f_{H}r_{HL}}{f_{L}\left(r_{HL}-1\right)}c_{V}\left(\chi\right)^{2}-f_{H}r\right)\psi\\ g_{L}+\left(\frac{f_{L}}{f_{H}\left(r_{HL}-1\right)}c_{V}\left(\chi\right)^{2}-f_{L}r\right)\psi& g_{L}+\left(-\frac{1}{r_{HL}-1}c_{V}\left(\chi\right)^{2}+f_{H}r\right)\psi\end{matrix}\right)$.

This matrix, in turn, can be shown to have a dominant eigenvalue of:

$R_{0}=H\left(\frac{1+\left(c_{V}\left(\chi\right)^{2}+r\right)\psi+\sqrt{1+2\left(c_{V}\left(\chi\right)^{2}-r\right)\psi+\left(c_{V}\left(\chi\right)^{2}+r\right)^{2}\psi^{2}}}{2}\right)$.

Although this expression remains a bit opaque, three important points can be noted: First, it is monotonically increasing with respect to *c_V_*(*χ*)^2^ and *r* (defined in table 2), representing measures of the relative degrees of risk heterogeneity and assortative mixing, respectively. Second, it is monotonically increasing with respect to *ψ*, and therefore monotonically decreasing with respect to *ω*. Finally, although three parameters (*f_H_*, *r_HL_*, and *m*) define the characteristics of behavioral heterogeneity apart from re-selection rate, *R*_0_ depends on these through only two derived parameters, *c_V_*(*χ*)^2^ and *r*.

Once *R*_0_ has been found, calculating the fraction of transmissions from EHI (*φ*) is relatively easy. Given *K* and its dominant eigenvalue (*R*_0_), we can easily solve for the corresponding eigenvector *v*. If *v* is scaled to sum to 1, then its entries represent the fraction of newly infected individuals who are in each of the initial states (*A_L,U_* and *A_H,U_*) in each generation during exponential growth. We can also decompose *K* into the sum of contributions to the NGM made by transmissions from EHI (*K*_1_) and contributions made by transmissions from chronic infection (*K*_2_). In the case of the model presented here, these matrices are:

$\begin{matrix} K_{1}=H_{1}\left(\begin{matrix} g_{H}+\left(\frac{r_{HL}}{r_{HL}-1}c_{V}\left(\chi\right)^{2}+f_{L}r\right)\psi_{1} & g_{H}+\left(-\frac{f_{H}r_{HL}}{f_{L}\left(r_{HL}-1\right)}c_{V}\left(\chi\right)^{2}-f_{H}r\right)\psi_{1} \\ g_{L}+\left(\frac{f_{L}}{f_{H}\left(r_{HL}-1\right)}c_{V}\left(\chi\right)^{2}-f_{L}r\right)\psi_{1} & g_{L}+\left(-\frac{1}{r_{HL}-1}c_{V}\left(\chi\right)^{2}+f_{H}r\right)\psi_{1} \end{matrix}\right) \\ K_{2}=H_{2}\left(\begin{matrix} g_{H}+\left(\frac{r_{HL}}{r_{HL}-1}c_{V}\left(\chi\right)^{2}+f_{L}r\right)\psi_{2} & g_{H}+\left(-\frac{f_{H}r_{HL}}{f_{L}\left(r_{HL}-1\right)}c_{V}\left(\chi\right)^{2}-f_{H}r\right)\psi_{2} \\ g_{L}+\left(\frac{f_{L}}{f_{H}\left(r_{HL}-1\right)}c_{V}\left(\chi\right)^{2}-f_{L}r\right)\psi_{2} & g_{L}+\left(-\frac{1}{r_{HL}-1}c_{V}\left(\chi\right)^{2}+f_{H}r\right)\psi_{2} \end{matrix}\right) \end{matrix}$.

The total number of transmissions per unit of infected population in one generation during exponential growth will then be ||*K*_1_*v*||_1_ (the sum of the two entries of the column vector *K*_1_*v*) from EHI, and ||*K*_2_*v*||_1_ from chronic infection. Consequently the fraction of transmissions from EHI is:

.

This can be expressed algebraically in terms of the original parameters, but the resulting equation is neither brief nor enlightening.

The formula for *R*_0_ is also useful in calculating the treatment rate required to achieve elimination of ongoing transmission (*τ_E_*): because elimination will occur precisely when *R*_0_ = 1, *τ_E_* can be found by solving for the value of *τ* that makes *R*_0_ = 1. Although it is technically possible to do this algebraically, it is simpler to do it numerically, and that is what we do. All code used is included at the end of the supplementary information.

§3.7 Construction of Figure 4

As noted in Table 2 in the main text, we parameterize the per-act transmissibility during EHI and chronic infection by *β*_2_ and *ζ*, or, more descriptively, the per-act transmissibility during chronic infection and the *relative* transmissibility during EHI (i.e. the ratio of *β*_1_ to *β*_2_). With this parameterization, *φ* depends on *ζ*, but not on *β*_2_, while the endemic prevalence depends on both. Consequently, we use the parameters of Supplementary Table 1 (except for *ω* and *ζ*), and for each value of *ω*, we first find a value for *ζ* that will result in a *φ* of 0.447. Then, using that value for *ζ*, we find a value for *β*_2_ that will result in an endemic prevalence of 0.2. With a full parameter set, we then find *R*_0_ and *τ_E_* using the methods detailed above.

§4 Supplementary Results

§4.1 Relationship between the basic reproduction number (*R*_0_), the fraction of transmission from early infection (*φ*), and the effective treatment rate required for elimination of ongoing transmission (*τ_E_*)

If we denote the basic reproduction number in the presence of diagnosis and treatment as *R*_0_(*τ*), then elimination will occur precisely when *R*_0_(*τ*) is reduced to 1, i.e.

$R_{0}\left(\tau_{E}\right)=1$.

Our previous equation for *R*_0_ easily extends to:

$R_{0}\left(\tau\right)=H\left(\tau\right)\left(\frac{1+\left(c_{V}\left(\chi\right)^{2}+r\right)\psi\left(\tau\right)+\sqrt{1+2\left(c_{V}\left(\chi\right)^{2}-r\right)\psi\left(\tau\right)+\left(c_{V}\left(\chi\right)^{2}+r\right)^{2}\psi\left(\tau\right)^{2}}}{2}\right)$.

In submodels without episodic risk (*ω* = 0), *ψ* = 1. Consequently, the above equation simplifies to:

$R_{0}\left(\tau\right)=H\left(\tau\right)\left(\frac{1+\left(c_{V}\left(\chi\right)^{2}+r\right)+\sqrt{1+2\left(c_{V}\left(\chi\right)^{2}-r\right)+\left(c_{V}\left(\chi\right)^{2}+r\right)^{2}}}{2}\right)$.

From this equation and the equations given in Supplementary Table 2, it follows that:

.

By solving this equation for $R_{0}\left(\tau_{E}\right)=1$, we obtain an equation for the effective treatment rate required to achieve elimination (*τ_E_*) that depends on *ζ* and the behavioral parameters (*f_H_*, *r_HL_*, and *m*) only through their effects on *R*_0_(0) (henceforth simply R_0_) and the fraction of transmissions from early infection (*φ*):

.

This result (depicted graphically in Supplementary Fig. 1) shows that *τ_E_* depends strongly on both *R*_0_ and *φ* (being monotonically increasing with respect to both). However, the effects of *R*_0_ are somewhat stronger than those of *φ* – if *φ* = 0, and *R*_0_ > 1, then *τ_E_* > 0, but if *φ* > 0, and *R*_0_ = 1, then *τ_E_* = 0.

As we discuss further below, this algebraic result does not hold in the full model, or in any submodel that includes episodic risk (*ω* ≠ 0). In the presence of episodic risk, behavioral parameters can have effects on *τ_E_* even when *R*_0_ and *φ* are both fixed. Nevertheless, the broader qualitative result, that *τ_E_* depends strongly on both *R*_0_ and *φ*, but somewhat more strongly on *R*_0_ than on *φ*, continues to apply.

§4.2 Effects of *ω* on *φ*

In the absence of assortative mixing, and during exponential growth, the average number of secondary transmissions from EHI per index case is $\left(1+c_{V}\left(\chi\right)^{2}\psi_{1}\right)H_{1}$, and the average number of secondary transmission during chronic infection per index case is $\left(1+c_{V}\left(\chi\right)^{2}\psi_{2}\right)H_{2}$. The fraction of transmissions (made by all individuals in a generation) that are from EHI is therefore:

.

For conceptual understanding, we are less interested in the exact value of *φ* at a particular *ω* (although this can be calculated from the equation above in a straightforward manner) than we are in the conditions under which *φ* is increasing or decreasing with respect to *ω*, i.e. we are interested in the sign of . This is easily shown to be the same as the sign of $\frac{\partial}{\partial\omega}\left(\frac{1+c_{V}\left(\chi\right)^{2}\psi_{1}}{1+c_{V}\left(\chi\right)^{2}\psi_{2}}\right)$. From this, and the equations given in Supplementary Table 2, it is a matter of straightforward algebra to show that $\frac{\partial}{\partial\omega}\left(\frac{1+c_{V}\left(\chi\right)^{2}\psi_{1}}{1+c_{V}\left(\chi\right)^{2}\psi_{2}}\right)$ is always decreasing, with a zero at:

$\omega=\sqrt{\nu_{1}\nu_{2}\left(1+c_{V}\left(\chi\right)^{2}\right)}$.

Thus, the fraction of transmissions from EHI is first increasing with respect to the re-selection rate, then decreasing, as noted in the main text. The maximum is attained when the re-selection rate is the product of the square root of the multiplicative boost to *R*_0_ due to *static* risk heterogeneity and the harmonic mean of the net rates of leaving (1) the untreated and acutely infected state and (2) the untreated and chronically infected state.

§4.3 Conversion of effective treatment rate to “actual” diagnosis rate

For a highly simplified calculation relating our concept of a single “effective treatment rate” to actual diagnosis rates, we will make the following simplifying assumptions:

1. Time from infection to diagnosis follows an exponential distribution with rate parameter *δ*, the diagnosis rate.
2. Time from diagnosis to entry into treatment follows an exponential distribution with rate parameter *ε*.
3. Time from entry into treatment to viral suppression follows an exponential distribution with rate parameter *σ*.
4. There is no virological failure after viral suppression.
5. The “effective treatment rate” *τ* for a treatment cascade as defined above is the rate parameter for an exponential distribution having the same mean as the actual distribution of times from infection to viral suppression, i.e.

.

None of these assumptions are actually true. Consequently, this is a poor model to use if the goal is to convert a specific effective treatment rate to a specific diagnostic rate. Nevertheless, it is useful for getting a qualitative sense of what the difference between two effective treatment rates required for elimination implies about the difference between the diagnostic rates required for elimination in their respective models. With that in mind, we proceed. A recent paper estimated the rate of initiating treatment following diagnosis as 77.2% within 3 months^3^. Combining this estimate with the above assumptions gives:

.

Crawford et al.^4^ report that the median time from initiating treatment to viral suppression for all patients was 1.03 years, and that for patients with optimal retention in care, it was 0.87 years. These figures give us (respectively):

$\begin{matrix} \sigma_{all}=\frac{-\ln\left(.5\right)}{1.03}\approx0.673 \\ \sigma_{optimal}=\frac{-\ln\left(.5\right)}{0.87}\approx0.797 \end{matrix}$.

Using the figure for all patients, *τ_E_* = 0.0635 implies that *δ_E_* = 0.0710. In contrast, *τ_E_* = 0.891 implies that the mean time from infection to viral suppression must be only 1.12 years. But even if we assume that perfect retention in care could be achieved for all patients, *σ* ≈ 0.797 implies that the mean time from entry into care to viral suppression is approximately 1.25 years, meaning that even instantaneous diagnosis and entry into care upon infection would not be sufficient to eliminate transmission through UT&T alone.

§4.4 Verification of qualitative results using an elaborated model of disease progression

In order to verify the robustness of the model to the relaxation of one of our simplifying assumptions, we tested a modified version in which we replaced our two-stage simplification of the natural history of HIV infection with a more realistic version of that natural history, based on the results of a study of HIV transmission in Lilongwe, Malawi^5^. In this version of the model, HIV progression consists of 9 stages, each of which has its own average duration, (relative) per-contact transmission rate, and identification as an acute stage or a chronic stage. The model used for the analysis of the Lilongwe data was Bayesian in nature; for our purposes, we used the posterior modes for each of the relevant parameters. We omitted the last of their 10 stages, corresponding to late AIDS, because they modeled this stage as having a transmission rate of 0, due to the cessation of sexual activity as the result of AIDS symptoms. Because our model does not include long-term partnerships, and only considers the sexually active population, individuals who have permanently ceased sexual activity are irrelevant to it. For consistency with our main model, we coded the progression rate for effectively treated individuals as being equal to the untreated rate for acute stages, and 0 for chronic stages. The accuracy of this approach is, for the purposes of this paper, irrelevant, as all of our analyses involve either the absence of effective treatment (when calculating the endemic prevalence prior to intervention) or conditions during exponential growth (when calculating the basic reproduction number or fraction of transmissions from EHI). Consequently, the fate of effectively treated individuals has no impact on any of our results. The initial parameters for each stage are given in Supplementary Table 3.

In order to perform analyses analogous to those we performed on our primary model, we calculated an average per-contact transmission rate for each of acute and chronic infection, by weighting the transmission rate for each stage by the expected time spent in that stage. We then treated these averages analogously to *β_1_* and *β_2_* in the main model, respectively. So, for example, where we allowed *β_1_* to vary in the main model, we allowed the transmission rates for all acute stages to vary, but required that the ratios between those transmission rates remain constant.

Figures corresponding to Figures 1, 2, 3, 4 in the main text, and Supplementary Figure 1, with no changes apart from the replacement of the main model with the elaborated model, are presented as Supplementary Figures 2, 3, 4, 5, and 6, respectively. It is readily seen that, although there are quantitative differences, these figures are all qualitatively the same as their main-model counterparts. This shows that our qualitative conclusions are robust to the relaxation of our simplifying assumption of a two-stage natural history of infection. Similarly, the only equations that we present in this paper that do not still hold for the elaborated model are those that explicitly indicate only two stages.

1. Newman, M. E. Mixing patterns in networks. *Phys. Rev. E* **67,** 026126 (2003).

2. Diekmann, O., Heesterbeek, J. A. P. & Roberts, M. G. The construction of next-generation matrices for compartmental epidemic models. *J. R. Soc. Interface* **7,** 873–885 (2010).

3. Torian, L. V. & Wiewel, E. W. Continuity of HIV-related medical care, New York City, 2005–2009: Do patients who initiate care stay in care? *AIDS Patient Care STDs* **25,** 79–88 (2011).

4. Crawford, T. N., Sanderson, W. T. & Thornton, A. Impact of Poor Retention in HIV Medical Care on Time to Viral Load Suppression. *J. Int. Assoc. Provid. AIDS Care JIAPAC* **13**, 242-249; DOI: 10.1177/2325957413491431 (2013).

5. Powers, K.A. *et al*. The role of acute and early HIV infection in the spread of HIV and implications from transmission prevention strategies in Lilongwe, Malawi: a modelling study. *Lancet* **378**, 256-268 (2011).

6. Volz, E. M. *et al.* HIV-1 transmission during early infection in men who have sex with men: a phylodynamic analysis. *PLoS Med.* **10,** e1001568; DOI: 10.1371/journal.pmed.1001568 (2013).

§5 Source Code

Introductory Note

All of the following separate files should be placed in the same directory. That directory’s parent directory *must* contain sub-directories named “pickles” and “graphics.” All figures generated by the following code will be written to the “graphics” directory. In addition to Python (version 3.4.1), the *NumPy* (version 1.9.0), *matplotlib* (version 1.4.0), and *SciPy* (version 0.14.0) libraries are required in order to run the following code. All figures were generated using the Anaconda distribution of Python, version 2.1.0 (64-bit).

FrozenDict.py

"""

Defines a frozen dict that inherits from Mapping and hashes equal to any other

FrozenDict with the same items.

Last modified 2014-06-09

"""

# NB: division is not used here, but I make it a policy to always import it.

from __future__ import division

import collections

class FrozenDict(collections.Mapping):

"""A frozen dict that hashes equal to any FrozenDict with the same items.

Behavior is the same as dict, except for immutability and hashability.

"""

def __init__(self, *args, **kwargs):

"""Create a FrozenDict and set its hash."""

self._dict = dict(*args, **kwargs)

"""The (mutable) dict that stores the items. Do not alter!"""

#self._hash = hash(tuple(sorted(self._dict.items())))

#"""This FrozenDict's hash value."""

def __iter__(self):

return iter(self._dict)

def __len__(self):

return len(self._dict)

def __getitem__(self, key):

return self._dict[key]

def __hash__(self):

return hash(tuple(sorted(self._dict.items())))#self._hash

def __repr__(self):

return 'FrozenDict(' + repr(self._dict) + ')'

general.py

"""General-purpose and utility functions.

Last updated 2014-06-09

"""

from __future__ import division

import itertools, os, imp, sys, copy #added copy

try:

import cPickle as pickle

except:

import pickle

import numpy as np

from FrozenDict import FrozenDict

#############

# Functions #

#############

def try_load_pickle(filename, function, *args, **kwargs):

"""Load a pickle if possible, otherwise create it.

Positional arguments:

filename -- location of the pickle to read/create

function -- function to call to generate the pickle

*args -- positional arguments to function

Keyword arguments:

**kwargs -- keyword arguments to function

Attempts to load a pickle stored in a file with name filename. If that file

does not exist, calls function(*args, **kwargs) and writes a pickle of the

return value to the file with name filename. Either way, returns the

resulting data.

"""

try:

with open(filename, 'rb') as loadfile:

dill = pickle.load(loadfile)

except IOError:

dill = function(*args, **kwargs)

with open(filename, 'wb') as dumpfile:

pickle.dump(dill, dumpfile)

return dill

def serialize_ranges(d):

"""Convert a dict of parameter ranges (as lists) to a list of FrozenDicts

each of which is one element of the Cartesian product of the original

ranges.

Positional arguments:

d -- dict mapping parameter names to lists of values that those parameters

take on

"""

flat = [[(name, value) for value in values] for name, values in d.items()]

return [FrozenDict(i) for i in itertools.product(*flat)]

def fit_parameter_sets(base_params, param_ranges, fit, *args, **kwargs):

"""Fit all parameter sets in the Cartesian product of param_ranges and

return a dict whose items are (FD: fit(based_params, FD, *args, **kwargs)),

where FD is an element of that Cartesian product in the form of a

FrozenDict.

Position arguments:

base_params -- dict of base parameters that are the same for all parameter

sets, unless overridden during fitting. If overridden, may be

starting points for a fitting algorithm.

param_ranges -- dict mapping parameter names to lists of values that those

parameters take on

fit -- function with signature fit(base_params, param_set, *args, **kwargs)

that returns the outcome of the desired fitting process. A return of

the form (params, equilibrium) is suggested but not required.

*args -- additional positional arguments for fit

Keyword arguments:

**kwargs -- keyword arguments for fit

"""

param_sets = serialize_ranges(param_ranges)

#base_params = FrozenDict(base_params)

base_params = copy.deepcopy(base_params) # somewhat less safe but necessary

N = len(param_sets)

print(N, ' sets to fit\n')

fits = {}

for i in range(N):

param_set = param_sets[i]

fits[param_set] = fit(base_params, param_set, *args, **kwargs)

sys.stdout.write(str(i) + ' ')

print('\n')

return fits

def dict_to_array(fits, param_names, param_ranges, extract_result, *args,

**kwargs):

"""Convert a dict mapping parameter sets to their fits to an n-dimensional

array of the outcomes of applying extract_results to those fits.

Positional arguments:

fits -- a dict whose items are (FD: fit), where FD is a dict of parameter

values and fit is a the outcome of applying a fitting function to

that set of parameter values.

param_names -- an iterable of parameter names, in the order that they should

be used to define axes of the returned array.

param_ranges -- a dict mapping the parameter names in param_names to

iterables of all the values they take on in fits.

extract_result -- a function with signature extract_result(fit, *args,

**kwargs) -> the outcome to be returned for that fit.

*args -- additional position arguments for extract_result

Keyword arguments:

**kwargs -- keyword arguments for extract_result

"""

s_pn = set(param_names)

s_prk = set(param_ranges.keys())

if len(param_names) != len(s_pn):

raise ValueError('Duplicated parameter names in ' + repr(param_names))

if s_pn != s_prk:

raise ValueError(repr(s_pn) + ' != ' + repr(s_prk))

lengths = [len(param_ranges[key]) for key in param_names]

print('Dimensions:', lengths, '\n')

n_params = len(lengths)

params = {}

a = np.zeros(lengths)

for index in itertools.product(*[range(length) for length in lengths]):

sys.stdout.write(str(index) + ' ')

for i in range(n_params):

param_name = param_names[i]

params[param_name] = param_ranges[param_name][index[i]]

args_ = fits[FrozenDict(params)] + args

a[index] = extract_result(*args_, **kwargs)

print('\n')

return a

def alert(n = 4):

"""Print the specified number of BEL characters to draw the user's attention

to the fact that execution has completed."""

sys.stdout.write('\a' * n)

model.py

"""Major functions for the model.

Note: description of "params" may be slightly off in a few docstrings.

Last modified 2015-02-14

"""

from __future__ import division

import imp, os, copy

import numpy as np

import scipy.integrate as spi

import general

from FrozenDict import FrozenDict

#############

# Functions #

#############

def fix_params(params):

if type(params) != dict:

params = dict(params)

params = copy.deepcopy(params) # protection

for key in ('progression_rate', 'progression_rate_treated', 'is_acute',

'transmissibility'):

params[key] = np.array(params[key])

return params

def odeint_func(y, t0, params): #t0 is required by odeint, but ignored

"""Calculate dy/dt based on the parameter set specified in params.

Positional arguments:

y -- size of the subpopulation in each compartment, expressed as a fraction

of the total population at the disease-free equilibrium. Order of

compartments is (S_H, S_L, A_H, C_H, A_L, C_L, A_HT, C_HT, A_LT, C_LT).

t0 -- nominally the time at which the derivative is being evaluated.

Required by scipy.integrate.odeint, but ignored.

params -- parameter set containing the following parameters:

gamma1 -- rate of transition from early to chronic infection

gamma2 -- excess mortality rate during untreated chronic infection

gamma2_T -- excess mortality rate during treated chronic infection

mu -- base mortality rate

chi -- average contact rate in the population at the DFE

rHL -- contact rate ratio between high- and low-risk

subpopulations

fH -- fraction of the population at high risk, at the DFE

beta2 -- per-contact transmission rate during untreated chronic

infection

beta1 -- per-contact transmission rate during untreated EHI

tau -- effective treatment rate

m -- fraction of contacts that are reserved for members of an

individual's own risk group

omega -- risk group re-selection rate

Returns dy/dt in the same order as y.

"""

progression_rate = params['progression_rate']

progression_rate_treated = params['progression_rate_treated']

transmissibility = params['transmissibility']

mu = params['mu']

chi = params['chi']

rHL = params['rHL']

fH = params['fH']

tau = params['tau']

m = params['m']

omega = params['omega']

fL = 1 - fH

chi_L = chi / (1 + (rHL - 1) * fH)

chi_H = rHL * chi_L

n = len(progression_rate)

H_S = y[0]

L_S = y[1]

H_U = y[2:(n + 2)]

L_U = y[(n + 2):(2 * n + 2)]

H_T = y[(2 * n + 2):(3 * n + 2)]

L_T = y[(3 * n + 2):]

H = H_S + sum(H_U) + sum(H_T)

L = L_S + sum(L_U) + sum(L_T)

FOI_from_L = sum(chi_L * transmissibility * L_U)

FOI_from_H = sum(chi_H * transmissibility * H_U)

FOI_L_only = FOI_from_L / (chi_L * L)

FOI_H_only = FOI_from_H / (chi_H * H)

FOI_general = (FOI_from_L + FOI_from_H) / (chi_L * L + chi_H * H)

FOI_L = m * FOI_L_only + (1 - m) * FOI_general

FOI_H = m * FOI_H_only + (1 - m) * FOI_general

H_transmissions = chi_H * FOI_H * H_S

L_transmissions = chi_L * FOI_L * L_S

H_births = fH * mu

L_births = fL * mu

H_U_progression = -1 * progression_rate * H_U

L_U_progression = -1 * progression_rate * L_U

H_T_progression = -1 * progression_rate_treated * H_T

L_T_progression = -1 * progression_rate_treated * L_T

for progression in (H_U_progression, L_U_progression,

H_T_progression, L_T_progression):

# -= works in place, and will threfore screw this up

progression[1:] = progression[1:] - progression[:-1]

H_treatment = tau * H_U

L_treatment = tau * L_U

#all migrations are expressed as net migration H to L

S_migration = omega * (fL * H_S - fH * L_S)

U_migration = omega * (fL * H_U - fH * L_U)

T_migration = omega * (fL * H_T - fH * L_T)

dH_S = H_births - H_transmissions - S_migration - mu * H_S

dL_S = L_births - L_transmissions + S_migration - mu * L_S

dH_U = H_U_progression - H_treatment - U_migration - mu * H_U

dH_U[0] += H_transmissions

dL_U = L_U_progression - L_treatment + U_migration - mu * L_U

dL_U[0] += L_transmissions

dH_T = H_T_progression + H_treatment - T_migration - mu * H_T

dL_T = L_T_progression + L_treatment + T_migration - mu * L_T

derivatives = np.concatenate(([dH_S], [dL_S], dH_U, dL_U, dH_T, dL_T))

return derivatives

def calculate_transition_matrix(params):

"""Calculate a transition matrix in the sense of Diekmann et al. 2010,

given a set of parameters.

Positional arguments:

params -- parameter set containing the following parameters:

gamma1 -- rate of transition from early to chronic infection

gamma2 -- excess mortality rate during untreated chronic infection

mu -- base mortality rate

fH -- fraction of the population at high risk, at the DFE

tau -- effective treatment rate

omega -- risk group re-selection rate

Returns a numpy.matrix with compartments in the order (A_H, C_H, A_L, C_L) or analagous thereto.

Treated compartments are not included because the individuals who enter

them are never again a source of transmissions, and are therefore

effectively removed from the population for the purpose of NGM methods.

This (along with the fact that all prevalence-fitting is done at an

effective treatment rate of 0) is the reason that none of our results

depend on the excess mortality rate for effectively treated individuals.

"""

progression_rate = params['progression_rate']

n = len(progression_rate)

tau = params['tau']

omega = params['omega']

fH = params['fH']

mu = params['mu']

fL = 1 - fH

nu = mu + progression_rate + tau

diagonal = -1 * np.concatenate((nu + fL * omega, nu + fH * omega))

Sigma = np.matrix(np.diag(diagonal))

Sigma[range(1, n), range(n - 1)] = progression_rate[:-1]

Sigma[range(n + 1, 2 * n), range(n, 2 * n - 1)] = progression_rate[:-1]

Sigma[:n,n:][range(n),range(n)] = fH * omega

Sigma[n:,:n][range(n),range(n)] = fL * omega

return Sigma

def calculate_transmission_matrix(params):

"""Calculate a transmission matrix in the sense of Diekmann et al. 2010,

given a set of parameters.

Positional arguments:

params -- parameter set containing the following parameters:

chi -- average contact rate in the population at the DFE

rHL -- contact rate ratio between high- and low-risk

subpopulations

fH -- fraction of the population at high risk, at the DFE

beta2 -- per-contact transmission rate during untreated chronic

infection

beta1 -- per-contact transmission rate during untreated EHI

m -- fraction of contacts that are reserved for members of an

individual's own risk group

Returns a numpy.matrix with compartments in the order (A_H, A_L, C_H, C_L).

Treated compartments are not included because the individuals who enter

them are never again a source of transmissions, and are therefore

effectively removed from the population for the purpose of NGM methods.

This (along with the fact that all prevalence-fitting is done at an

effective treatment rate of 0) is the reason that none of our results

depend on the excess mortality rate for effectively treated individuals.

"""

chi = params['chi']

transmissibility = params['transmissibility']

n = len(transmissibility)

m = params['m']

rHL = params['rHL']

fH = params['fH']

fL = 1 - fH

chi_L = chi / (1 + (rHL - 1) * fH)

chi_H = rHL * chi_L

g_H = fH * rHL / (1 + fH * (rHL - 1))

g_L = fL / (1 + fH * (rHL - 1))

H_to_H = chi_H * (g_H + m * g_L) * transmissibility

H_to_L = chi_H * (1 - m) * g_L * transmissibility

L_to_H = chi_L * (1 - m) * g_H * transmissibility

L_to_L = chi_L * (g_L + m * g_H) * transmissibility

T = np.matrix(np.zeros((2 * n, 2 * n)))

T[0,:] = np.concatenate((H_to_H, L_to_H))

T[n,:] = np.concatenate((H_to_L, L_to_L))

return T

def calculate_NGM(params):

"""Calculate a next-generation matrix (NGM) in the sense of Diekmann et

al. 2010, given a set of parameters.

Positional arguments:

params -- parameter set containing the following parameters:

gamma1 -- rate of transition from early to chronic infection

gamma2 -- excess mortality rate during untreated chronic infection

gamma2_T -- excess mortality rate during treated chronic infection

mu -- base mortality rate

chi -- average contact rate in the population at the DFE

rHL -- contact rate ratio between high- and low-risk

subpopulations

fH -- fraction of the population at high risk, at the DFE

beta2 -- per-contact transmission rate during untreated chronic

infection

beta1 -- per-contact transmission rate during untreated EHI

tau -- effective treatment rate

m -- fraction of contacts that are reserved for members of an

individual's own risk group

omega -- risk group re-selection rate

Returns a numpy.matrix with compartments in the order (A_H, A_L). Note that

this is the NGM (K), not the NGM with large domain (K_L)

"""

Sigma = calculate_transition_matrix(params)

T = calculate_transmission_matrix(params)

KL = -T * Sigma.I

n = len(params['transmissibility'])

K = KL[(0, n), :][:, (0, n)]

return K

def calculate_dominant_eigenvalue_and_vector(M):

"""Calculate the dominant eigenvalue and eigenvector of a real numpy.matrix.

Requires that the dominant eigenvalue be real.

Positional arguments:

M -- a numpy.matrix

Returns:

eigenvalue -- the dominant eigenvalue

eigenvector -- the corresponding eigenvector

Raises a ValueError if either is not real (after processing by

numpy.real_if_close, to deal with round-off error).

"""

eigenvalues, eigenvectors = np.linalg.eig(M)

i = np.argmax([eigenvalue.real for eigenvalue in eigenvalues])

# np.real_if_close always returns an array for some reason

eigenvalue = np.real_if_close(eigenvalues[i]).sum()

eigenvector = np.real_if_close(eigenvectors[:, i])

try:

assert (np.isreal(eigenvalue) and

all(np.isreal(vi) for vi in eigenvector))

return eigenvalue, eigenvector

except AssertionError:

raise ValueError(str(eigenvalue) + ' and/or ' + str(eigenvector) +

'is not real, in matrix M with eigenvalues {lambda} and' +

'eigenvectors {v} where\nM =\n' + str(M) + '\n\n{lambda} =' +

str(eigenvalues) + '\n\n{v} =\n' + str(eigenvectors) + '\n')

def calculate_instantaneous_distribution(params):

"""Calculate the dominant eigenvector of the Jacobian matrix of the

linearization of the transmission system (specified by params) at the

disease-free equilibrium.

Positional arguments:

params -- parameter set containing the following parameters:

gamma1 -- rate of transition from early to chronic infection

gamma2 -- excess mortality rate during untreated chronic infection

gamma2_T -- excess mortality rate during treated chronic infection

mu -- base mortality rate

chi -- average contact rate in the population at the DFE

rHL -- contact rate ratio between high- and low-risk

subpopulations

fH -- fraction of the population at high risk, at the DFE

beta2 -- per-contact transmission rate during untreated chronic

infection

beta1 -- per-contact transmission rate during untreated EHI

tau -- effective treatment rate

m -- fraction of contacts that are reserved for members of an

individual's own risk group

omega -- risk group re-selection rate

"""

J = (calculate_transition_matrix(params) +

calculate_transmission_matrix(params))

return calculate_dominant_eigenvalue_and_vector(J)[1]

def calculate_phi_and_R0(params):

"""Calculate the next-generation matrix (NGM) R0 of Diekmann et al. (2010),

and, under the same set of assumptions, the fraction of transmissions during

exponential growth that are made by transmitters in EHI.

Positional arguments:

params -- parameter set containing the following parameters:

gamma1 -- rate of transition from early to chronic infection

gamma2 -- excess mortality rate during untreated chronic infection

gamma2_T -- excess mortality rate during treated chronic infection

mu -- base mortality rate

chi -- average contact rate in the population at the DFE

rHL -- contact rate ratio between high- and low-risk

subpopulations

fH -- fraction of the population at high risk, at the DFE

beta2 -- per-contact transmission rate during untreated chronic

infection

beta1 -- per-contact transmission rate during untreated EHI

tau -- effective treatment rate

m -- fraction of contacts that are reserved for members of an

individual's own risk group

omega -- risk group re-selection rate

Returns:

phi -- fraction of transmissions from EHI

R0 -- basic reproduction number, respectively

"""

K = calculate_NGM(params)

R0, distribution = calculate_dominant_eigenvalue_and_vector(K)

test_R0_sign(params, R0)

distribution /= distribution.sum()

Sigma = calculate_transition_matrix(params)

time_spent = -Sigma.I

T = calculate_transmission_matrix(params)

is_acute = params['is_acute']

n = len(is_acute)

is_acute = np.tile(is_acute, 2) # for high- and low-risk

transmissions_1 = (T[(0, n), :][:, is_acute] *

time_spent[is_acute, :][:, (0, n)] * distribution).sum()

transmissions_2 = (T[(0, n), :][:, ~is_acute] *

time_spent[~is_acute, :][:, (0, n)] * distribution).sum()

np.testing.assert_almost_equal(transmissions_1 + transmissions_2, R0)

return transmissions_1 / R0, R0

def test_R0_sign(params, R0):

"""Tests that R0 - 1 has the correct sign, based on the output of

odeint_func.

Positional arguments:

params -- parameter set containing the following parameters:

gamma1 -- rate of transition from early to chronic infection

gamma2 -- excess mortality rate during untreated chronic infection

gamma2_T -- excess mortality rate during treated chronic infection

mu -- base mortality rate

chi -- average contact rate in the population at the DFE

rHL -- contact rate ratio between high- and low-risk

subpopulations

fH -- fraction of the population at high risk, at the DFE

beta2 -- per-contact transmission rate during untreated chronic

infection

beta1 -- per-contact transmission rate during untreated EHI

tau -- effective treatment rate

m -- fraction of contacts that are reserved for members of an

individual's own risk group

omega -- risk group re-selection rate

R0 -- putative basic reproduction number to be tested

Returns sign(R0 - 1) if both agree, and raises ValueError otherwise.

"""

epsilon = 1e-9 # how large of an infected population to use

infected_deltas_zero_tolerance = 1e-12 # cutoffs are fairly arbitrary

R0_zero_tolerance = 1e-6

distribution = calculate_instantaneous_distribution(params)

distribution /= sum(distribution)

n2 = len(distribution)

y = ((1 - epsilon) * np.array(disease_free_equilibrium(params)) + epsilon *

np.concatenate((np.zeros(2), np.array(distribution.flat),

np.zeros(n2))))

delta_y = np.array(odeint_func(y, 0, params))

infected_deltas = delta_y[2:(2 + n2)]

try:

if all(infected_deltas < 0) and R0 < 1:

return -1

if all(infected_deltas > 0) and R0 > 1:

return 1

if (all(abs(infected_deltas) < infected_deltas_zero_tolerance) and

abs(R0 - 1) < R0_zero_tolerance):

return 0

except TypeError:

raise TypeError(infected_deltas)

raise Exception(R0, distribution, y, delta_y, infected_deltas)

def calculate_R0(params):

"""Calculate the next-generation matrix (NGM) R0 of Diekmann et al. (2010).

Positional arguments:

params -- parameter set containing the following parameters:

gamma1 -- rate of transition from early to chronic infection

gamma2 -- excess mortality rate during untreated chronic infection

gamma2_T -- excess mortality rate during treated chronic infection

mu -- base mortality rate

chi -- average contact rate in the population at the DFE

rHL -- contact rate ratio between high- and low-risk

subpopulations

fH -- fraction of the population at high risk, at the DFE

beta2 -- per-contact transmission rate during untreated chronic

infection

beta1 -- per-contact transmission rate during untreated EHI

tau -- effective treatment rate

m -- fraction of contacts that are reserved for members of an

individual's own risk group

omega -- risk group re-selection rate

"""

K = calculate_NGM(params)

R0 = calculate_dominant_eigenvalue_and_vector(K)[0]

test_R0_sign(params, R0)

return R0

def calculate_betas(params):

progression_rate = params['progression_rate']

mu = params['mu']

is_acute = params['is_acute']

fraction_surviving_previous_stage = (progression_rate[:-1] /

(progression_rate[:-1] + mu))

fraction_surviving = np.concatenate(([1],

fraction_surviving_previous_stage.cumprod()))

person_time = fraction_surviving / (progression_rate + mu)

contribution = person_time * params['transmissibility']

beta1 = sum(contribution[is_acute]) / sum(person_time[is_acute])

beta2 = sum(contribution[~is_acute]) / sum(person_time[~is_acute])

return beta1, beta2

def calculate_equilibrium_betas(params, equilibrium):

transmissibility = params['transmissibility']

n = len(transmissibility)

chi = params['chi']

rHL = params['rHL']

fH = params['fH']

fL = 1 - fH

chi_L = chi / (1 + (rHL - 1) * fH)

chi_H = rHL * chi_L

high = equilibrium[2:(n + 2)]

low = equilibrium[(n + 2):(2 * n + 2)]

contacts = high * chi_H + low * chi_L

contribution = contacts * transmissibility

is_acute = params['is_acute']

beta1 = sum(contribution[is_acute]) / sum(contacts[is_acute])

beta2 = sum(contribution[~is_acute]) / sum(contacts[~is_acute])

beta = sum(contribution) / sum(contacts)

return beta1, beta2, beta

def calculate_zeta(params):

beta1, beta2 = calculate_betas(params)

return beta1 / beta2

def force_zeta(params):

params = copy.deepcopy(params)

beta1, beta2 = calculate_betas(params)

zeta_0 = beta1 / beta2

zeta = params['zeta']

params['transmissibility'][params['is_acute']] *= zeta / zeta_0

return params

def fit_phi(params):

"""Alter parameters beta2, beta1, and/or zeta in order to make the fraction

of transmissions from EHI during exponential growth equal target_phi,

without altering other parameters, and find the endemic equilibrium.

Does not preserve R0.

Tacitly assumes that params['transmissibility'] and params['is_acute'] are

such as to generate a well-defined and non-zero zeta at the start of

fitting.

Positional arguments:

params -- parameter set containing the following parameters:

gamma1 -- rate of transition from early to chronic infection

gamma2 -- excess mortality rate during untreated chronic infection

gamma2_T -- excess mortality rate during treated chronic infection

mu -- base mortality rate

chi -- average contact rate in the population at the DFE

rHL -- contact rate ratio between high- and low-risk

subpopulations

fH -- fraction of the population at high risk, at the DFE

beta2 -- per-contact transmission rate during untreated chronic

infection

beta1 -- per-contact transmission rate during untreated EHI

tau -- effective treatment rate

m -- fraction of contacts that are reserved for members of an

individual's own risk group

omega -- risk group re-selection rate

target_phi -- desired fraction of transmissions from EHI during

exponential growth

Returns:

params -- updated and fitted paramater set

equilibrium -- the corresponding endemic equilibrium

"""

tolerance = 1e-5

maximum_iterations = 1000

params = fix_params(params)

transmissibility = params['transmissibility'] # this works because ndarray

is_acute = params['is_acute']

target_phi = params['target_phi']

if target_phi == 0:

params['zeta'] = 0

params['transmissibility'][params['is_acute']] = 0

return params

if target_phi == 1:

params['zeta'] = float('inf')

params[~params['is_acute']] = 0

return params

zeta = calculate_zeta(params)

upper = float('inf')

lower = 0

phi = calculate_phi_and_R0(params)[0]

i = 0

while abs(phi - target_phi) > tolerance:

if i > maximum_iterations:

raise Exception(

str(maximum_iterations) +

' iterations were not sufficent to find a value for zeta' +

' that would result in a fraction of transmissions from' +

' EHI of ' + str(target_phi) + ' within a tolerance of ' +

str(tolerance) + '.\nMost recent results:\nzeta = ' +

str(params['zeta']) + '\nphi = ' + str(phi) + '\n\n')

if phi < target_phi:

lower = zeta

if upper == float('inf'):

zeta *= 2

transmissibility[is_acute] *= 2

else:

zeta = (upper + lower)/2

transmissibility[is_acute] *= zeta / lower

else:

upper = zeta

zeta = (lower + upper)/2

transmissibility[is_acute] *= zeta / upper

phi = calculate_phi_and_R0(params)[0]

i += 1

equilibrium = find_equilibrium(params)

new_zeta = calculate_zeta(params)

np.testing.assert_almost_equal(zeta, new_zeta) # just as a sanity check

params['zeta'] = zeta # just for record-keeping

return params, equilibrium

def fit_prevalence_and_phi(base_params, param_set):

"""Alter parameters beta2, beta1, and/or zeta in order to make the fraction

of transmissions from EHI during exponential growth equal target_phi and the

endemic prevalence equal target_prevalence, without altering other

parameters, and find the endemic equilibrium.

Positional arguments:

base_params, param_set -- combined into a derived argument params with

values from param_set overwriting those from

base_params in the case of a conflict

params -- parameter set containing the following parameters:

gamma1 -- rate of transition from early to chronic infection

gamma2 -- excess mortality rate during untreated chronic infection

gamma2_T -- excess mortality rate during treated chronic infection

mu -- base mortality rate

chi -- average contact rate in the population at the DFE

rHL -- contact rate ratio between high- and low-risk

subpopulations

fH -- fraction of the population at high risk, at the DFE

beta2 -- per-contact transmission rate during untreated chronic

infection

beta1 -- per-contact transmission rate during untreated EHI

tau -- effective treatment rate

m -- fraction of contacts that are reserved for members of an

individual's own risk group

omega -- risk group re-selection rate

target_phi -- desired fraction of transmissions from EHI during

exponential growth

target_prevalence -- desired endemic prevalence

Returns:

params -- updated and fitted paramater set

equilibrium -- the corresponding endemic equilibrium

"""

params = dict(base_params)

params.update(param_set)

if 'zeta' in param_set:

params = force_zeta(params)

params, equilibrium = fit_phi(params)

params, equilibrium = fit_prevalence(params, {})

return params, equilibrium

def disease_free_equilibrium(params):

"""Return the disease-free equilibrium distribution of population between

compartments.

Positional arguments:

params -- parameter set containing the following parameters:

fH - fraction of the population at high risk, at the DFE

"""

n = len(params['transmissibility'])

return np.array([params['fH'], 1 - params['fH']] + (4 * n) * [0])

def find_prevalence(y):

"""Calculate prevalence of infection.

Positional arguments:

y -- size of the subpopulation in each compartment, expressed as a fraction

of the total population at the disease-free equilibrium. Order of

compartments is (S_H, S_L, A_H, C_H, A_L, C_L, A_HT, C_HT, A_LT, C_LT).

"""

P = sum(y)

I = sum(y[2:])

return I/P

def find_infecteds(y):

"""Calculate number of infected individuals, as a fraction

of the total population at the disease-free equilibrium.

Positional arguments:

y -- size of the subpopulation in each compartment, expressed as a fraction

of the total population at the disease-free equilibrium. Order of

compartments is (S_H, S_L, A_H, C_H, A_L, C_L, A_HT, C_HT, A_LT, C_LT).

"""

I = sum(y[2:])

return I

def find_high_risk_infecteds(y):

"""Calculate number of infected individuals at high-risk, as a fraction

of the total population at the disease-free equilibrium.

Positional arguments:

y -- size of the subpopulation in each compartment, expressed as a fraction

of the total population at the disease-free equilibrium. Order of

compartments is (S_H, S_L, A_H, C_H, A_L, C_L, A_HT, C_HT, A_LT, C_LT).

"""

n = (len(y) - 2) / 4

H_S = y[0]

L_S = y[1]

H_U = y[2:(n + 2)]

L_U = y[(n + 2):(2 * n + 2)]

H_T = y[(2 * n + 2):(3 * n + 2)]

L_T = y[(3 * n + 2):]

I_H = sum(H_U) + sum(H_T)

return I_H

def find_infecteds_over_high_risk_infecteds(y):

"""Calculate the fraction of infected individuals who are in the high-risk

group.

Positional arguments:

y -- size of the subpopulation in each compartment, expressed as a fraction

of the total population at the disease-free equilibrium. Order of

compartments is (S_H, S_L, A_H, C_H, A_L, C_L, A_HT, C_HT, A_LT, C_LT).

"""

I = find_infecteds(y)

I_H = find_high_risk_infecteds(y)

try:

return I / I_H

except ZeroDivisionError:

if I == 0:

return float('nan')

else:

return float('inf')

def trivial_fit(base_params, param_set):

"""Update a parameter set and calculate the endemic equilibrium, with no

actual fitting.

Positional arguments:

base_params, param_set -- dicts to be combined into a new dict "params".

Where the same key exists in both, the value from

param_set will be used.

params -- parameter set containing the following parameters:

gamma1 -- rate of transition from early to chronic infection

gamma2 -- excess mortality rate during untreated chronic infection

gamma2_T -- excess mortality rate during treated chronic infection

mu -- base mortality rate

chi -- average contact rate in the population at the DFE

rHL -- contact rate ratio between high- and low-risk

subpopulations

fH -- fraction of the population at high risk, at the DFE

beta2 -- per-contact transmission rate during untreated chronic

infection

beta1 -- per-contact transmission rate during untreated EHI

tau -- effective treatment rate

m -- fraction of contacts that are reserved for members of an

individual's own risk group

omega -- risk group re-selection rate

target_phi -- desired fraction of transmissions from EHI during

exponential growth

target_prevalence -- desired endemic prevalence

Returns:

params -- updated paramater set

equilibrium -- the corresponding endemic equilibrium

"""

params = fix_params(base_params)

params.update(param_set)

if 'zeta' in param_set:

params = force_zeta(params)

return params, find_equilibrium(params)

def find_equilibrium(params, tolerance = 1e-8, maximum_iterations = 1e4,

T = 100, mxstep = 0):

"""Find the endemic equilibrium, given a parameter set.

Positional arguments:

params -- parameter set containing the following parameters:

gamma1 -- rate of transition from early to chronic infection

gamma2 -- excess mortality rate during untreated chronic infection

gamma2_T -- excess mortality rate during treated chronic infection

mu -- base mortality rate

chi -- average contact rate in the population at the DFE

rHL -- contact rate ratio between high- and low-risk

subpopulations

fH -- fraction of the population at high risk, at the DFE

beta2 -- per-contact transmission rate during untreated chronic

infection

beta1 -- per-contact transmission rate during untreated EHI

tau -- effective treatment rate

m -- fraction of contacts that are reserved for members of an

individual's own risk group

omega -- risk group re-selection rate

target_phi -- desired fraction of transmissions from EHI during

exponential growth

target_prevalence -- desired endemic prevalence

Keyword arguments:

tolerance -- maximum value for the sum of all ||dy_i/dt|| in order to

consider the system "at equilibrium" (default 1e-8)

maximum_iterations -- maximum number of calls to scipy.integrate.odeint that

should be made without attaining equilibrium before

giving up (default 1e4)

T -- number of units of time (years) per call to scipy.integrate.odeint

(default 100)

mxstep -- maximum number of steps per odeint call (default 0 - i.e. solver-

defined)

"""

if calculate_R0(params) <= 1:

try:

return disease_free_equilibrium(params)

except:

print('R0 < 0; error in disease_free_equilibrium(params)')

raise

y = starting_point(params)

i = 0

derivatives = odeint_func(y, 0, params)

deviation = sum([abs(x) for x in derivatives])

while deviation > tolerance or min(y) < 0:

T *= 2

if i > maximum_iterations:

raise Exception(str(maximum_iterations) + ' iterations were ' +

'not sufficent to achieve equilibrium within' +

'a tolerance of ' + str(tolerance) +

'.\nMost recent results:\nSub-population sizes:'

+ str(y) + '\nDerivatives:' + str(derivatives) +

'\n\n')

output = spi.odeint(odeint_func, y, (0, T), (params,), mxstep = mxstep)

y_ = output[-1, :]

while min(y_) < 0:

T /= 2

output = spi.odeint(odeint_func, y, (0, T), (params,),

mxstep = mxstep)

y_ = output[-1, :]

y = y_

i += 1

derivatives = odeint_func(y, 0, params)

deviation = sum([abs(x) for x in derivatives])

return y

def fit_prevalence(base_params, param_set, tolerance = 1e-4,

maximum_iterations = 1000):

"""Alter parameter beta2 (and beta1, if defined explicitly) to make the

endemic prevalence equal target_prevalence, without altering other

parameters, and find the endemic equilibrium.

Positional arguments:

base_params, param_set -- combined into a derived argument params with

values from param_set overwriting those from

base_params in the case of a conflict

params -- parameter set containing the following parameters:

gamma1 -- rate of transition from early to chronic infection

gamma2 -- excess mortality rate during untreated chronic infection

gamma2_T -- excess mortality rate during treated chronic infection

mu -- base mortality rate

chi -- average contact rate in the population at the DFE

rHL -- contact rate ratio between high- and low-risk

subpopulations

fH -- fraction of the population at high risk, at the DFE

beta2 -- per-contact transmission rate during untreated chronic

infection

beta1 -- per-contact transmission rate during untreated EHI

tau -- effective treatment rate

m -- fraction of contacts that are reserved for members of an

individual's own risk group

omega -- risk group re-selection rate

target_prevalence -- desired endemic prevalence

Keyword arguments:

tolerance -- maximum absolute difference between the observed and

target prevalences (default 1e-4)

maximum_iterations -- maximum number of binary search steps to make before

giving up (default 1000)

Returns:

params -- updated and fitted paramater set

equilibrium -- the corresponding endemic equilibrium

"""

params = fix_params(base_params)

params.update(param_set)

if 'zeta' in param_set:

params = force_zeta(params)

scale = 1 #how much to scale params['transmissibility'] by

upper = float('inf')

lower = 0

equilibrium = find_equilibrium(params)

try:

prevalence = find_prevalence(equilibrium)

except:

print('first', equilibrium)

raise

target_prevalence = base_params['target_prevalence']

i = 0

while abs(prevalence - target_prevalence) > tolerance:

if i > maximum_iterations:

raise Exception(str(maximum_iterations) + ' iterations were' +

'not sufficent to find a value for beta2' + ' that would' +

'result in a prevalence of ' + str(target_prevalence) +

' within a tolerance of ' + str(tolerance) +

'.\nMost recent results:\nbeta2 = ' + str(params['beta2']) +

'\nPrevalence = ' + str(prevalence) + '\n\n')

if prevalence < target_prevalence:

lower = scale

if upper == float('inf'):

scale *= 2

else:

scale = (upper + lower)/2

params['transmissibility'] *= scale / lower

else:

upper = scale

scale = (upper + lower)/2

params['transmissibility'] *= scale / upper

equilibrium = find_equilibrium(params)

try:

prevalence = find_prevalence(equilibrium)

except:

print('second', equilibrium)

raise

i += 1

return params, equilibrium

def find_tau_E(params, _, tolerance = 1e-4, maximum_iterations = 1000):

"""Find the effective treatment rate required to achieve elimination.

Positional arguments:

params -- parameter set containing the following parameters:

gamma1 -- rate of transition from early to chronic infection

gamma2 -- excess mortality rate during untreated chronic infection

gamma2_T -- excess mortality rate during treated chronic infection

mu -- base mortality rate

chi -- average contact rate in the population at the DFE

rHL -- contact rate ratio between high- and low-risk

subpopulations

fH -- fraction of the population at high risk, at the DFE

beta2 -- per-contact transmission rate during untreated chronic

infection

beta1 -- per-contact transmission rate during untreated EHI

tau -- effective treatment rate

m -- fraction of contacts that are reserved for members of an

individual's own risk group

omega -- risk group re-selection rate

target_phi -- desired fraction of transmissions from EHI during

exponential growth

target_prevalence -- desired endemic prevalence

_ -- in actual usage, the endemic equilibrium population distribution.

Ignored.

Keyword arguments:

tolerance -- maximum absoulte value for R0 - 1 (default 1e-4)

maximum_iterations -- maximum number of calls to boolean search steps to

make before giving up (default 1000)

T -- number of units of time (years) per call to scipy.integrate.odeint

Returns:

tau -- the effective treatment rate required to achieve elimination

"""

params = fix_params(params)

R0 = calculate_R0(params)

i = 0

debug_list = [(params['tau'], R0)]

lower = 0

upper = float('inf')

while abs(R0 - 1) > tolerance:

if i > maximum_iterations:

for i in debug_list:

print(i)

raise Exception(str(maximum_iterations) +

' iterations were not sufficent to find a value for tau' +

' that would result in an R0 of 1 ' +

' within a tolerance of ' + str(tolerance) +

'.\nMost recent results:\ntau = ' +

str(params['tau']) + '\nR0 = ' + str(R0) + '\n\n')

if R0 > 1:

lower = params['tau']

if upper == float('inf'):

if params['tau'] >= 1:

params['tau'] *= 2

else:

params['tau'] = 1

else:

params['tau'] = (upper + params['tau'])/2

else:

upper = params['tau']

params['tau'] = (lower + params['tau'])/2

R0 = calculate_R0(params)

i += 1

debug_list.append((params['tau'], R0))

return params['tau']

def starting_point(params):

n = len(params['transmissibility'])

return np.array((2 * n + 2) * [1 / (2 * n + 2)] + (2 * n) * [0])

#############

# Constants #

#############

simple_base_params = FrozenDict(

set_name = 'simple',

progression_rate = (1, 1/(1.89 * 5 - 1)),

progression_rate_treated = (1, 0),

is_acute = (True, False),

mu = 1./40.28,

fH = 0.1,

m = 0,

rHL = 21,

omega = 1,

chi = 20,

transmissibility = (0.02, 0.002), # just a starting point

tau = 0, #likewise

target_phi = 0.447,

target_prevalence = 0.2,

)

weekly = 52.1775

Powers_base_params = FrozenDict(

set_name = 'Powers',

progression_rate = (weekly, weekly, weekly, weekly, weekly / 16.6,

1/1.9, 1/1.9, 1/1.9, 1/0.9), # late AIDS omitted

progression_rate_treated = (weekly, weekly, weekly, weekly, weekly / 16.6,

0, 0, 0, 0),

is_acute = (True, True, True, True, True,

False, False, False, False),

mu = 1./40.28,

fH = 0.1,

m = 0,

rHL = 21,

omega = 1,

chi = 20,

transmissibility = (0.003, 0.03, 0.04, 0.03, 0.02,

0.0007, 0.0007, 0.0007, 0.006),

tau = 0, #likewise

target_phi = 0.447,

target_prevalence = 0.2,

)

heatmaps.py

"""Common plotting function for Figure 1 and Figure 2 and plotting function

for Supplementary Figure 1."""

import matplotlib.pyplot as plt

import mpl_toolkits.axes_grid1

import matplotlib.colors

def outcome_heatmaps(datasets, xticks, yticks, xticklabels, yticklabels,

bar_labels, xlabel, ylabel, filename = None):

"""Plot 1 or more heatmaps, stacked vertically.

Positional arguments:

datasets -- sequence of arrays, each of which is to be turned into a heatmap

xticks -- sequence of floats

yticks -- sequence of floats

xticklabels -- list of strings

yticklabels -- list of strings

bar_labels -- sequence of strings, to be used to label the colorbar for

each heatmap

xlabel -- string

ylabel -- string

filename -- string or None. If filename is a string, it represents the name

of the file that the plot should be saved in. If None, it

indicates that the plot should be displayed on-screen.

(default None)

"""

plt.clf()

N = len(datasets)

fig, axes = plt.subplots(N, 1, True, True, figsize = (4, 3 * N))

for i in range(N):

ax = axes[i]

values = datasets[i]

try:

ax.tick_params(labelsize = 8)

except:

print(fig, axes, ax)

print(bar_labels[i], ':', values.min(), values.max())

if bar_labels[i] == r'$\tau_E$':

image = ax.pcolor(values, vmax = 0.7)

else:

image = ax.pcolor(values)

ax.set_xticks(xticks)

ax.set_yticks(yticks)

ax.set_xticklabels(xticklabels)

ax.set_yticklabels(yticklabels)

if i == N - 1:

ax.set_xlabel(xlabel)

ax.set_ylabel(ylabel)

ax_ = mpl_toolkits.axes_grid1.make_axes_locatable(ax)

cax = ax_.append_axes("right", size="10%", pad=0.05)

bar = fig.colorbar(image, cax=cax, orientation = 'vertical')

bar.ax.tick_params(labelsize = 8)

bar.set_label(bar_labels[i], rotation = 'vertical')

panel_label = '(' + chr(ord('a') + i) + ')'

ax.text(-3, 14, panel_label)

if filename is None:

plt.show()

else:

plt.savefig(filename, bbox_inches = 'tight')

plt.close()

def heatmap(array, min_, max_, xticks, yticks, xticklabels, yticklabels,

bar_label, title = '', xlabel = '', ylabel = '', filename = None):

"""Plot a heatmap.

Positional arguments:

array -- array to be turned into a heatmap

min_ -- minimum value used in defining the mapping of values to colors

max_ -- maximum value used in defining the mapping of values to colors

xticks -- sequence of floats

yticks -- sequence of floats

xticklabels -- list of strings

yticklabels -- list of strings

bar_label -- strings, to be used to label the colorbar for the heatmap

title -- title of the heatmap (default '')

xlabel -- string (default '')

ylabel -- string (default '')

filename -- string or None. If filename is a string, it represents the name

of the file that the plot should be saved in. If None, it

indicates that the plot should be displayed on-screen.

(default None)"""

plt.clf()

fig, ax = plt.subplots()

try:

ax.tick_params(labelsize = 8)

except:

print(fig, ax)

raise

image = ax.pcolor(array, norm = matplotlib.colors.Normalize(min_, max_))

ax.set_xticks(xticks)

ax.set_yticks(yticks)

ax.set_xticklabels(xticklabels)

ax.set_yticklabels(yticklabels)

ax.set_xlabel(xlabel)

ax.set_ylabel(ylabel)

ax.set_title(title, size = 15)

bar = fig.colorbar(image, orientation = 'horizontal')

bar.ax.tick_params(labelsize = 8)

bar.set_label(bar_label)

if filename is None:

plt.show()

else:

plt.savefig(filename, bbox_inches = 'tight')

plt.close()

Figure 1.py

"""Plots endemic prevalence and R0 vs. re-selection rate and relative

transmissibility during EHI, with total transmission potential fixed.

Last modified 2015-02-14

"""

import os, imp

import numpy as np

import general, model, heatmaps

from FrozenDict import FrozenDict

################################

# Date for pickles and figures #

################################

date = '2015-02-14'

##################

# Body functions #

##################

def homogeneous_potential(params):

"""Calculate the total transmission potential."""

#copied block from model.calculate_betas

progression_rate = params['progression_rate']

mu = params['mu']

is_acute = params['is_acute']

fraction_surviving_previous_stage = (progression_rate[:-1] /

(progression_rate[:-1] + mu))

fraction_surviving = np.concatenate(([1],

fraction_surviving_previous_stage.cumprod()))

person_time = fraction_surviving / (progression_rate + mu)

contribution = person_time * params['transmissibility']

#end copied block

return sum(contribution)

def homogeneous_potential_fit(base_params, param_set):

"""Update base_params with values from param_set, and adjust beta1 and beta2

in order to have the same total transmission potential as before the update.

"""

params = model.fix_params(base_params)

target_potential = homogeneous_potential(params)

params.update(param_set)

params = model.force_zeta(params) #new line

potential = homogeneous_potential(params)

params['transmissibility'] *= target_potential / potential

equilibrium = model.find_equilibrium(params)

return params, equilibrium

def obtain_fits(base_params):

"""Load or generate parameter sets and equilibria for a range of

re-selection rates and relative transmissibilities, with total transmission

potential fixed."""

base_params = base_params.copy()

param_ranges = {'omega': 10 ** np.arange(-1, 1.1, .125),

'zeta': np.arange(1, 9.1, .5)}

#fixing a single point of commonality, in the lower left corners

base_params = model.fit_prevalence(base_params,

dict(omega = 0.1, zeta = 1))[0]

set_name = base_params['set_name']

results_dict_pickle_filename = ('../pickles/Figure 1 -- ' + set_name + ' ' +

date + '.pickle')

results_dict = general.try_load_pickle(results_dict_pickle_filename,

general.fit_parameter_sets,

base_params, param_ranges,

homogeneous_potential_fit)

return results_dict

def plot_outcome_heatmaps(fits, set_name):

"""Plot heatmaps depicting endemic prevalence and R0 vs. re-selection rate

and relative transmissibility during EHI."""

param_ranges = {'omega': 10 ** np.arange(-1, 1.1, .125),

'zeta': np.arange(1, 9.1, .5)}

R0s = general.dict_to_array(fits, ['omega', 'zeta'], param_ranges,

lambda params, eq: model.calculate_R0(params))

Ps = general.dict_to_array(fits, ['omega', 'zeta'], param_ranges,

lambda params, eq: model.find_prevalence(eq))

yticklabels = param_ranges['omega'][::8]

xticklabels = param_ranges['zeta'][::4]

yticks = np.arange(0, len(param_ranges['omega']), 8)+0.5

xticks = np.arange(0, len(param_ranges['zeta']), 4)+0.5

bar_labels = (r'$R_0$', r'$P$')

filename = ('../graphics/Figure 1 -- ' + set_name + ' ' + date + '.jpg')

heatmaps.outcome_heatmaps((R0s, Ps), xticks, yticks, xticklabels,

yticklabels, bar_labels, r'$\zeta$',

r'$\omega$ ($year^{-1}$)',

filename)

################

# Main section #

################

if __name__ == '__main__':

try:

for master_params in (dict(model.simple_base_params),

dict(model.Powers_base_params)):

fits = obtain_fits(master_params)

print('Fits done')

plot_outcome_heatmaps(fits, master_params['set_name'])

finally:

general.alert()

#$R_0$ : 1.00812436541 3.82618425086

#$P$ : 0.00765617787851 0.347925389965

#$R_0$ : 1.09686265326 4.10282355149

#$P$ : 0.0828990884919 0.37606570712 1

Figure 2.py

"""Plots fractions of transmissions from EHI during exponential growth, R0, and

effective treatement rate required to achieve elimination vs. re-selection rate

and relative transmissibility during EHI, with endemic prevalence fixed.

Last modified 2015-02-14

"""

import os, imp

import numpy as np

import general, model, heatmaps

from FrozenDict import FrozenDict

################################

# Date for pickles and figures #

################################

date = '2015-02-14'

##################

# Body functions #

##################

def obtain_fits(base_params):

"""Load or generate parameter sets and equilibria for a range of

re-selection rates and relative transmissibilities, with endemic prevalence

fixed."""

base_params = base_params.copy()

param_ranges = {'omega': 10 ** np.arange(-1, 1.1, .125),

'zeta': np.arange(1, 9.1, .5)}

set_name = base_params['set_name']

results_dict_pickle_filename = ('../pickles/Figure 2 -- ' + set_name + ' ' +

date + '.pickle')

results_dict = general.try_load_pickle(results_dict_pickle_filename,

general.fit_parameter_sets,

base_params, param_ranges,

model.fit_prevalence)

return results_dict

def plot_outcome_heatmaps(fits, set_name):

"""Plot heatmaps depicting fractions of transmissions from EHI during

exponential growth, R0, and effective treatement rate required to achieve

elimination vs. re-selection rate and relative transmissibility during EHI.

"""

param_ranges = {'omega': 10 ** np.arange(-1, 1.1, .125),

'zeta': np.arange(1, 9.1, .5)}

R0s = general.dict_to_array(fits, ['omega', 'zeta'], param_ranges,

lambda params, eq: model.calculate_R0(params))

phis = general.dict_to_array(fits, ['omega', 'zeta'], param_ranges,

lambda params, eq:

model.calculate_phi_and_R0(params)[0])

tau_Es = general.dict_to_array(fits, ['omega', 'zeta'], param_ranges,

model.find_tau_E)

yticklabels = param_ranges['omega'][::8]

xticklabels = param_ranges['zeta'][::4]

yticks = np.arange(0, len(param_ranges['omega']), 8)+0.5

xticks = np.arange(0, len(param_ranges['zeta']), 4)+0.5

bar_labels = (r'$\tau_E$ ($year^{-1}$)', r'$R_0$', r'$\phi$')

filename = ('../graphics/Figure 2 -- ' + set_name + ' ' + date + '.jpg')

heatmaps.outcome_heatmaps((tau_Es, R0s, phis), xticks, yticks, xticklabels,

yticklabels, bar_labels, r'$\zeta$',

r'$\omega$ ($year^{-1}$)',

filename)

################

# Main section #

################

if __name__ == '__main__':

try:

for master_params in (dict(model.simple_base_params),

dict(model.Powers_base_params)):

fits = obtain_fits(master_params)

print('Fits done')

beta1s = list()

beta2s = list()

betas = list()

for params, equilibrium in fits.values():

beta1, beta2, beta = model.calculate_equilibrium_betas(

params, equilibrium)

beta1s.append(beta1)

beta2s.append(beta2)

betas.append(beta)

print('Beta1s (min, max, mean):', min(beta1s), max(beta1s),

np.mean(beta1s))

print('Beta2s (min, max, mean):', min(beta2s), max(beta2s),

np.mean(beta2s))

print('Betas (min, max, mean):', min(betas), max(betas),

np.mean(betas))

plot_outcome_heatmaps(fits, master_params['set_name'])

f = lambda zeta, omega: model.find_tau_E(

*fits[FrozenDict(zeta=zeta,omega=omega)])

for zeta, omega in ((9, 0.1), (9, 10), (1, 0.1)):

print(zeta, omega, ':', f(zeta, omega))

finally:

general.alert()

#$\tau_E$ ($year^{-1}$) : 0.0398864746094 1.4677734375

#$R_0$ : 1.26639385011 3.33689231947

#$\phi$ : 0.163444512732 0.756828358316

#9 0.1 : 1.4677734375

#9 10 : 0.100830078125

#1 0.1 : 0.442138671875

#$\tau_E$ ($year^{-1}$) : 0.0526733398438 2.6240234375

#$R_0$ : 1.27075551293 3.76514537571

#$\phi$ : 0.0860691665741 0.66050600911

#9 0.1 : 2.6240234375

#9 10 : 0.117431640625

#1 0.1 : 0.4619140625

Figure 3.py

"""Plots prevalence vs. R0 for a variety of related models.

Last modified 2015-02-14

"""

import os, imp

import numpy as np

import matplotlib.pyplot as plt

import model, general

################################

# Date for pickles and figures #

################################

date = '2015-02-14'

#############

# Functions #

#############

def get_params(model_name, base_params):

"""Retrieve and modify the base parameter set appropriate for each model."""

params = model.fix_params(base_params) #dict(model.base_params)

if model_name == 'EA':

params['m'] = 0.5

return params

if model_name == 'EP':

return params

if model_name == 'SA':

params['m'] = 0.5

params['omega'] = 0

return params

if model_name == 'SP':

params['omega'] = 0

return params

if model_name == 'BH':

params['omega'] = 0

params['rHL'] = 1

return params

raise ValueError(model_name)

def get_line(model_name):

"""Return the appropriate line quality for plotting for the given model."""

if model_name == 'EA':

return 'r-'

if model_name == 'EP':

return 'r:'

if model_name == 'SA':

return 'b-'

if model_name == 'SP':

return 'b:'

if model_name == 'BH':

return 'k-'

raise ValueError(model_name)

#############

# Main Code #

#############

if __name__ == '__main__':

try:

for master_params in (dict(model.simple_base_params),

dict(model.Powers_base_params)):

plt.clf()

model_names = ('BH', 'SP', 'SA', 'EP', 'EA')

for model_name in model_names:

params = get_params(model_name, master_params)

R0_base = model.calculate_R0(params)

chi_over_R0 = params['chi'] / R0_base

R0_range = np.arange(1, 20.05, .1)

R0s = np.zeros(200) #in theory unnecessary, but a good precaution

Ps = np.zeros(200)

for i in range(200):

params['chi'] = (i + 1)/10 * chi_over_R0

R0s[i] = model.calculate_R0(params)

Ps[i] = model.find_prevalence(

model.find_equilibrium(params))

plt.plot(R0s, Ps, get_line(model_name), label = model_name)

plt.plot((0, 20), (0.2, 0.2), 'k--')

plt.xlabel(r'Basic Reproduction Number ($R_0$)')

plt.ylabel('Endemic Prevalence (P)')

plt.xlim(0, 25)

plt.legend()

set_name = params['set_name']

plt.savefig('../graphics/Figure 3 -- ' + set_name + ' ' + date +

'.jpg')

#print(plt.axis())

plt.close()

finally:

general.alert()

Figure 4.py

"""

Plots R0 and tau_E vs. omega with phi fixed.'

Last modified 2015-02-14

"""

from __future__ import division

import os, imp

import numpy as np

import matplotlib.pyplot as plt

import general, model

################################

# Date for pickles and figures #

################################

date = '2015-02-14'

##################

# Body functions #

##################

def obtain_double_fits(base_params):

"""Load or generate parameter sets and equilibria for a range of

re-selection rates, with endemic prevalence and fraction of early

transmissions fixed."""

base_params = model.fix_params(base_params)

param_ranges = {'omega': 10 ** np.arange(-1, 1.1, .1)}

set_name = base_params['set_name']

results_dict_pickle_filename = ('../pickles/Figure 4 -- ' + set_name + ' ' +

date + '.pickle')

results_dict = general.try_load_pickle(results_dict_pickle_filename,

general.fit_parameter_sets,

base_params, param_ranges,

model.fit_prevalence_and_phi)

return results_dict

def plot_double_curves(fits, set_name):

"""Plot R0 and tau_E vs. re-selection rate."""

param_ranges = {'omega': 10 ** np.arange(-1, 1.1, .1)}

R0s = general.dict_to_array(fits, ['omega'], param_ranges,

lambda params, eq: model.calculate_R0(params))

tau_Es = general.dict_to_array(fits, ['omega'], param_ranges,

model.find_tau_E)

_, ax = plt.subplots()

ax.set_xlabel(r'Re-selection rate ($\omega$) ($year^{-1}$)')

ax.set_ylabel(r'Effective treatment rate required to achieve elimination ' +

r'($\tau_E$)' + '\n($year^{-1}$)')

line1 = ax.semilogx(param_ranges['omega'], tau_Es, 'b-',

label = r'$\tau_E$')

ax2 = ax.twinx()

ax2.set_ylabel(r'Basic reproduction number ($R_0$)')

line2 = ax2.semilogx(param_ranges['omega'], R0s, 'b--', label = r'$R_0$')

lines = line1 + line2

labels = [l.get_label() for l in lines]

ax.legend(lines, labels)

filename = ('../graphics/Figure 4 -- ' + set_name + ' ' + date + '.jpg')

plt.savefig(filename, bbox_inches = 'tight')

print('R0 range: (', min(R0s), ',', max(R0s), ')')

print('tau_E range: (', min(tau_Es), ',', max(tau_Es), ')')

################

# Main section #

################

if __name__ == '__main__':

try:

for master_params in (dict(model.simple_base_params),

dict(model.Powers_base_params)):

fits = obtain_double_fits(master_params)

print('Fits done')

set_name = master_params['set_name']

plot_double_curves(fits, set_name)

finally:

general.alert()

#R0 range: ( 1.26742262563 , 3.27509189732 )

#tau_E range: ( 0.0634765625 , 0.89111328125 )

#R0 range: ( 1.2725825217 , 3.75845580355 )

#tau_E range: ( 0.0936279296875 , 2.4912109375)

Supplementary Figure 1.py

"""Generates a heatmap plotting tau_E vs. R0 and fraction of early transmissions

under behavioral homogeneity (or static risk heterogeneity and proportional

mixing).

Last modifed 2015-02-14

"""

from __future__ import division

import imp, os

import numpy as np

import general, model, heatmaps

################################

# Date for pickles and figures #

################################

date = '2015-02-14'

#############

# Functions #

#############

#From Figure 1

def calculate_contributions(params):

#copied block from model.calculate_betas

progression_rate = params['progression_rate']

mu = params['mu']

fraction_surviving_previous_stage = (progression_rate[:-1] /

(progression_rate[:-1] + mu))

fraction_surviving = np.concatenate(([1],

fraction_surviving_previous_stage.cumprod()))

person_time = fraction_surviving / (progression_rate + mu)

contribution = person_time * params['transmissibility']

#end copied block

return contribution

def calculate_tau_E(base_params, param_set):

"""Calculate the effective treatment rate required to achieve elimination.

Also checks its algebraic result against the numerical result from

model.find_tau_E, thereby increasing confidence in both."""

#now only does the algebra for the simple model

global max_difference

params = model.fix_params(base_params)

params.update(param_set)

if 'zeta' in param_set:

params = model.force_zeta(params)

R0 = params['R0']

phi = params['phi']

contribution = calculate_contributions(params)

is_acute = params['is_acute']

transmissibility = params['transmissibility']

target_1 = R0 * phi

current_1 = params['chi'] * sum(contribution[is_acute])

transmissibility[is_acute] *= target_1 / current_1

target_2 = R0 * (1 - phi)

current_2 = params['chi'] * sum(contribution[~is_acute])

transmissibility[~is_acute] *= target_2 / current_2

approximated = model.find_tau_E(params, None)

if base_params['set_name'] == 'simple':

nu1 = params['mu'] + params['progression_rate'][0]

nu2 = params['mu'] + params['progression_rate'][1]

b = nu1 + nu2 - R0 * phi * nu1

c = -(R0 - 1) * nu1 * nu2

tau_E = (-b + np.sqrt(b ** 2 - 4 * c)) / 2

difference = abs(approximated - tau_E)

try:

if difference > max_difference:

max_difference = difference

print(difference, tau_E, approximated)

except NameError:

max_difference = difference

print(difference, tau_E, approximated)

else:

tau_E = approximated

return (tau_E,)

def plot_tau_E_vs_R0_and_phi(base_params):

"""Plot a heatmap of effective treatment rate required to achieve

elimination vs. R0 and fraction of early transmissions."""

param_ranges = {'R0': np.arange(1, 6.05, .5), 'phi': np.arange(0, 1.01, .1)}

set_name = base_params['set_name']

results_dict_pickle_filename = ('../pickles/Supplementary Figure 1 -- ' +

set_name + ' ' + date + '.pickle')

results_dict = general.try_load_pickle(results_dict_pickle_filename,

general.fit_parameter_sets,

base_params, param_ranges,

calculate_tau_E)

def identity(*x):

if len(x) == 1:

return x[0]

else:

raise ValueError(x)

results = general.dict_to_array(results_dict, ['R0', 'phi'], param_ranges,

identity)

max_ = results.max()

min_ = results.min()

print(min_, max_)

min_ = np.floor(min_)

max_ = .7

xticklabels = param_ranges['phi'][::2]

yticklabels = param_ranges['R0'][::2]

xticks = np.arange(0, len(param_ranges['phi']), 2)+0.5

yticks = np.arange(0, len(param_ranges['R0']), 2)+0.5

bar_label = r'$\tau_{E}$'

filename = ('../graphics/Supplementary Figure 1 - ' + set_name + ' ' + date +

'.jpg')

heatmaps.heatmap(results, min_, max_, xticks, yticks, xticklabels,

yticklabels, bar_label, bar_label + r' vs. $R_0$ and $\phi$',

r'$\phi$', r'$R_0$', filename)

#############

# Main code #

#############

if __name__ == '__main__':

try:

for master_params in (dict(model.simple_base_params),

dict(model.Powers_base_params)):

master_params['m'] = 0

master_params['omega'] = 0

master_params['rHL'] = 1

plot_tau_E_vs_R0_and_phi(master_params)

finally:

general.alert()

#(0.0, 5.1241310824230384)

#(0.0, 14.7734375)
